# Multi-omics of a model bacterial consortium deciphers details of chitin decomposition in soil

**DOI:** 10.1128/mbio.00404-25

**Published:** 2025-05-30

**Authors:** Ryan McClure, Albert Rivas-Ubach, Kim K. Hixson, Yuliya Farris, Marci Garcia, Robert Danczak, Michelle Davison, Vanessa L. Paurus, Janet K. Jansson

**Affiliations:** 1 Biological Sciences Division, Pacific Northwest National Laboratory 6865, Richland, Washington, USA; 2 Earth and Biological Sciences Directorate, Pacific Northwest National Laboratory 6865, Richland, Washington, USA; 3 Environmental and Molecular Sciences Division (EMSL), Pacific Northwest National Laboratory 6865, Richland, Washington, USA; 4 Institute of Forest Sciences (ICIFOR-INIA), Spanish National Research Council (CSIC) 383204 , Madrid, Spain; 5 Physical and computational Sciences Division, Pacific Northwest National Laboratory 6865, Richland, Washington, USA; 6 Advanced Computing, Mathematics, and Data Division, Pacific Northwest National Laboratory 6865, Richland, Washington, USA; University of Washington School of Medicine, Seattle, Washington, USA

**Keywords:** soil microbiome, model soil consortium-2, chitin, chitin decomposition, multi-omics, metabolomics, metatranscriptomics, metaproteomics

## Abstract

Soil microorganisms interact to carry out decomposition of complex organic carbon and nitrogen compounds, such as chitin, but the high diversity and complexity of the soil microbiome and habitat have posed a challenge to elucidating such interactions. Here, we sought to address this challenge by analysis of a model soil consortium (MSC-2) consisting of eight soil bacterial species. Our aim was to elucidate the specific roles of the member species during chitin metabolism. Samples were collected from MSC-2 incubated in chitin-enriched soil over 3 months. Multi-omics was used to understand how the community composition, transcripts, proteins, and chitin decomposition shifted over time. The data clearly and consistently revealed a temporal shift during chitin decomposition with defined contributions by individual species. A *Streptomyces* genus member (sp001905665) was a key player in early steps of chitin decomposition, with other MSC-2 members being central in carrying out later steps. These results illustrate how multi-omics applied to a defined consortium untangles the interactions between soil microorganisms.

**IMPORTANCE:**

Although soil microorganisms carry out decomposition of organic matter in soil, the details are unclear due to the complexity of the soil microbiome and the heterogeneity of the soil habitat. Understanding carbon decomposition is of vital importance to determine how the soil carbon cycle functions. This is especially important with regard to understanding the fertility of soils and their ability to support plant growth. To overcome these challenges, we investigated in considerable detail a model soil community during its decomposition of a typical soil organic molecule—chitin. By using a multi-omics approach, we were able to decipher community interactions during chitin breakdown. This information provides a basis for understanding how more complex soil microbial communities interact in nature.

## INTRODUCTION

Prior work has shown that the cycling of soil organic carbon is driven in large part by a complex network of interactions among soil microbial species (1). However, the individual interactions between specific soil microorganisms during carbon decomposition are largely unknown. This knowledge gap is compounded by the heterogeneity of the environment, the high soil microbial diversity (2), as well as functional redundancy that results in several different species contributing to decomposition in a complex web of interactions (3).

Organic polymers, such as lignin, cellulose, and chitin, can be difficult to decompose compared to simpler monomers (4). While their abundance means that enzymes to decompose them are widespread in the environment (5), they require extracellular processing and hydrolysis before the resulting smaller subunits can be imported into the cell for metabolic processing. Here, we focused on filling this knowledge gap for the decomposition of chitin, the second-most abundant organic compound on the planet, after cellulose (6). In soil, chitin is introduced as part of insect exoskeletons and fungal cell walls (6). Studies in aquatic systems (7, 8) have revealed that chitin can be degraded by direct enzymatic hydrolysis of the glycosidic bonds linking N-acetylglucosamine units. Alternatively, chitin may be first deacetylated to chitosan and then deaminated. However, much less is known about the individual steps involved in chitin decomposition in soil or how species may interact to decompose chitin as a community.

Recently, a model soil consortium (MSC-1) was developed through natural evolution of the soil microbiome using chitin as a substrate and dilution and passaging to reduce the complexity (9). Subsequently, eight species were isolated from MSC-1 and combined to comprise a synthetic community named Model Soil Consortium-2 (MSC-2) (10, 11). MSC-2 includes the following species/strains: *Streptomyces* sp001905665 strain 001, *Neorhizobium tomejilense* strain 005, *Dyadobacter* sp. strain 007, *Sphingopyxis* sp. strain 008, *Ensifer adhaerens* strain 011, *Variovorax beijingensis* strain 012*, Sinorhizobium meliloti* strain 014, and *Rhodococcus* sp003130705 strain 016 (numbered based on the order of isolate collection in a previous publication (10)).

MSC-2 was previously characterized during chitin decomposition in liquid culture (10, 11). Genome models revealed that the different species had different enzymatic capabilities for chitin degradation. Although the data obtained were valuable in defining contributions of MSC-2 species to chitin decomposition in the liquid medium, they did not necessarily reflect interactions that could occur in soil. Soil provides a solid matrix and microniches that can facilitate or impede reactions between community members (12, 13). The MSC-2 isolates were originally obtained from an arid grassland field soil. Therefore, we hypothesized that the addition of the eight strains back to their original source soil would best approximate how the species would interact as chitin is decomposed over time in the soil environment in nature. Because each of the eight species has been genome-sequenced, we were able to integrate a variety of -omics data (amplicon sequencing, metatranscriptomics, and metaproteomics) to follow each strain and their expression of RNA transcripts and proteins during incubation in the natural soil environment over 12 weeks with data collected and compared at four discrete time points. In addition, we deployed metabolomics to follow chitin degradation products. These data enabled us to map the pathway for chitin decomposition by a defined community of bacteria in soil. This study is one of the first to determine and define specific interspecies microbial metabolic dynamics in a soil environment over time using a multi-omics approach. The approach that we demonstrate here, using a well-characterized, reduced complexity community examined under soil conditions, will be a useful approach for exploring how specific members of the soil microbiome cycle carbon in different soil ecosystems and how carbon cycling is impacted by a changing environment.

## RESULTS

### Dynamics of MSC-2 during incubation of soil with chitin

The eight MSC-2 strains were incubated in sterile field soil containing chitin (500 ppm) as a substrate (Fig. S1). The MSC-2 inoculum was added at two doses: 10^9^ and 10^8^ cells/gram of soil (Table S1). DNA, RNA, proteins, and metabolites were collected after 0, 4, 8, and 12 weeks of incubation in soil. All data comparisons and conclusions were focused on these four soil time points.

There was an initial increase in DNA yield after inoculation, suggesting cell growth: 19-fold with 10^8^ cells per gram and threefold with 10^9^ cells per gram (Fig. S2). The increase in DNA yield could reflect growth on the supplemented chitin substrate and/or cryptic growth on dead soil biomass. Changes in the difference in increases in DNA yield at different inoculation levels may also reflect the carrying capacity of the soil for microbial biomass. When comparing 4 to 8 weeks of incubation, DNA levels increased by an additional twofold in soil inoculated with 10^8^ cells but decreased by 30% in soils inoculated with 10^9^ cells. Subsequently, the DNA levels decreased by 46% and 40% in soils inoculated with 10^8^ cells or 10^9^ cells per gram of soil, respectively, suggesting that cell death occurred as chitin was consumed (supported by our metabolomics data below).

**Fig 1:**
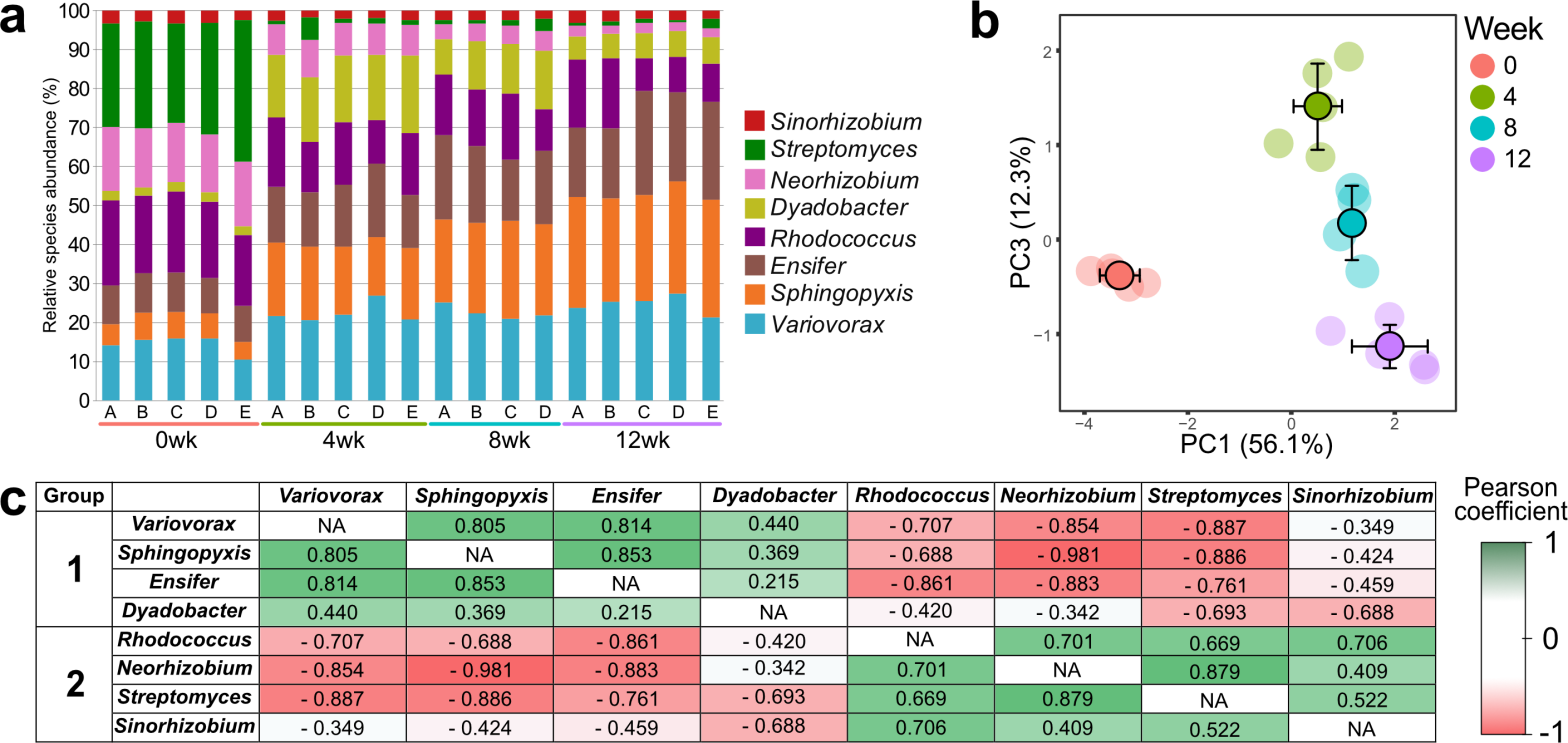
Model Soil Consortium-2 (MSC-2) species dynamics during chitin decomposition. (**a**) Relative species abundance in five replicate soil samples (**a–e**), over 0, 4, 8, and 12 weeks of incubation using 16S data. (**b**) Principal component (PC) 1 vs PC2 of the score plot of the principal component analysis (PCA) showing community composition shifts over incubation periods (0 = 0 weeks, red; 4 = 4 weeks, green; 8 = weeks, blue; 12 = 12 weeks, purple). Faded dots represent the individual samples, and deep colored dots with black outlines represent the average value (error bars ± SD) for each of the incubation periods on the plane defined by the PC1 vs PC2. (**c**) Pearson coefficients of positive (green) and negative (red) interactions between MSC-2 species during 12 weeks of incubation.

16S rRNA gene sequencing revealed that the vast majority of recovered nucleic acids originated from the MSC-2 inoculum, with negligible background DNA from other species (Table S2), and minimal relic DNA in uninoculated soil controls (Fig. S2), highlighting the fact that the soil was successfully sterilized at the start of the experiment. The species representation in the MSC-2 inoculum was relatively even at the beginning of the experiment; thus, all species began at the same “starting line” (Fig. S3). The species composition of MSC-2 changed during the incubations with both the high (Fig. 1) and low inoculum doses (Fig. S4) based on our 16S data. Because both doses had similar taxonomic compositions at the start of the experiment, and because samples with 10^9^ cells per gram of soil had higher yields of DNA (as well as RNA and protein, see below), we focused on the higher dose for subsequent analyses. Note that only genus designations are used below for each of the MSC-2 species to simplify the text. All 16S rRNA data from all samples are in Table S3.

The MSC-2 species composition shifted initially when comparing 0 to 4 weeks of incubation at 20°C (Fig. 1). Note that this shift also included a 5-day preincubation at 4°C. The preincubation was included to mimic conditions required for future studies of MSC-2 under conditions on the International Space Station (ISS), which requires time to transport samples from the ground to the ISS (see Materials and Methods). Therefore, chitin decomposition may have been initiated prior to the first sampling point. Because the parent chitin compound cannot be measured using our instrumentation, we focused on microbial shifts in abundance that occurred during processing of chitin degradation intermediates.

At subsequent time points, the MSC-2 species composition continued to shift with chitin metabolism (Fig. 1b), but the magnitude of the change was less than that seen when comparing 0 to 4 weeks of incubation (Table S4). Species that increased in relative abundance from 0 to 4 weeks were *Dyadobacter, Variovorax, Sphingopyxis,* and *Ensifer,* while those that decreased in abundance were *Streptomyces, Neorhizobium,* and *Sinorhizobium* (Fig. 1a; Table S4). When comparing 4 weeks to 12 weeks of incubation, *Sphingopyxis* and *Ensifer* continued to increase in relative abundance, while *Neorhizobium* continued to decline in relative abundance. By contrast, *Dyadobacter*, while initially showing a large increase in the relative abundance from 0 to 4 weeks, subsequently declined. All the shifts described above were statistically significant with a *P*-value of <0.05. We include absolute amounts of 16S rRNA reads from each species in Fig. S5 and one-way ANOVAs on the score coordinates of all PCA plots in Table S5.

To define which MSC-2 members were interacting with each other (either negatively or positively), the individual species abundances were compared across time points and replicates. This analysis showed that *Ensifer* was strongly positively correlated with both *Variovorax* and *Sphingopyxis*, with the latter two species also strongly correlated with each other (Fig. 1c). In addition, *Streptomyces* and *Neorhizobiu*m were strongly correlated with each other. By contrast, *Ensifer, Variovorax,* and *Sphingopyxis* were all negatively correlated with *Streptomyces* and *Neorhizobium*. Finally, *Rhodococcus* was negatively correlated with *Ensifer,* and *Sinorhizobium* was negatively correlated with *Dyadobacter*. All correlations described above were significant with an adjusted *P*-value of <0.05. These data revealed that the eight species could be clustered into two groups that had similar correlation profiles: each group with four species (Fig. 1c).

### Metatranscriptomics of MSC-2 in soil during chitin decomposition

**Fig 2:**
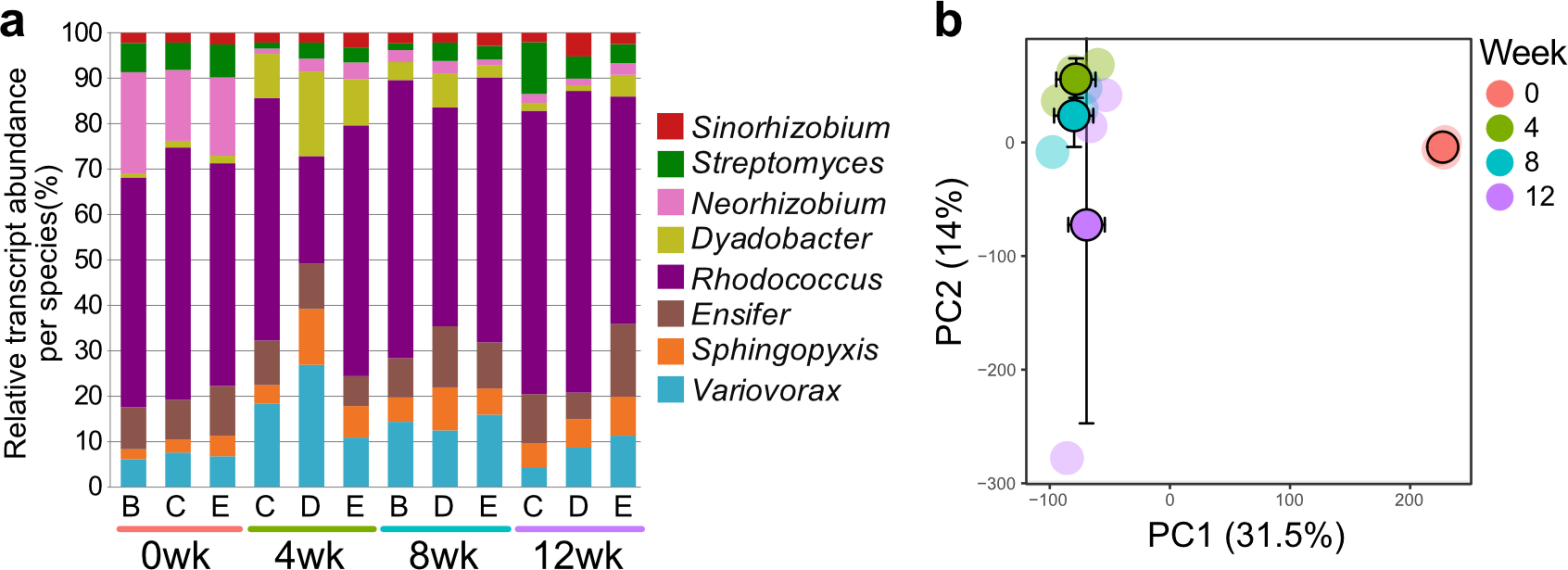
Gene expression by MSC-2 consortium members during the 12 week incubation with chitin in soil. (**a**) Relative transcript abundance of MSC-2 species over 1, 4, 8, and 12 weeks of incubation. Letters below each column correspond to one of three sample replicates. (**b**) PC1 vs PC2 of the score plot of the PCA showing shifts in MSC-2 consortium transcripts over weeks of incubation: 0 weeks (red), 4 weeks (green), 8 weeks (blue), and 12 weeks (purple). Faded dots represent the individual samples, and deep colored dots with black outlines represent the average value (error bars ± SD) for each of the incubation periods on the plane defined by the PC1 vs PC2.

**Fig 3:**
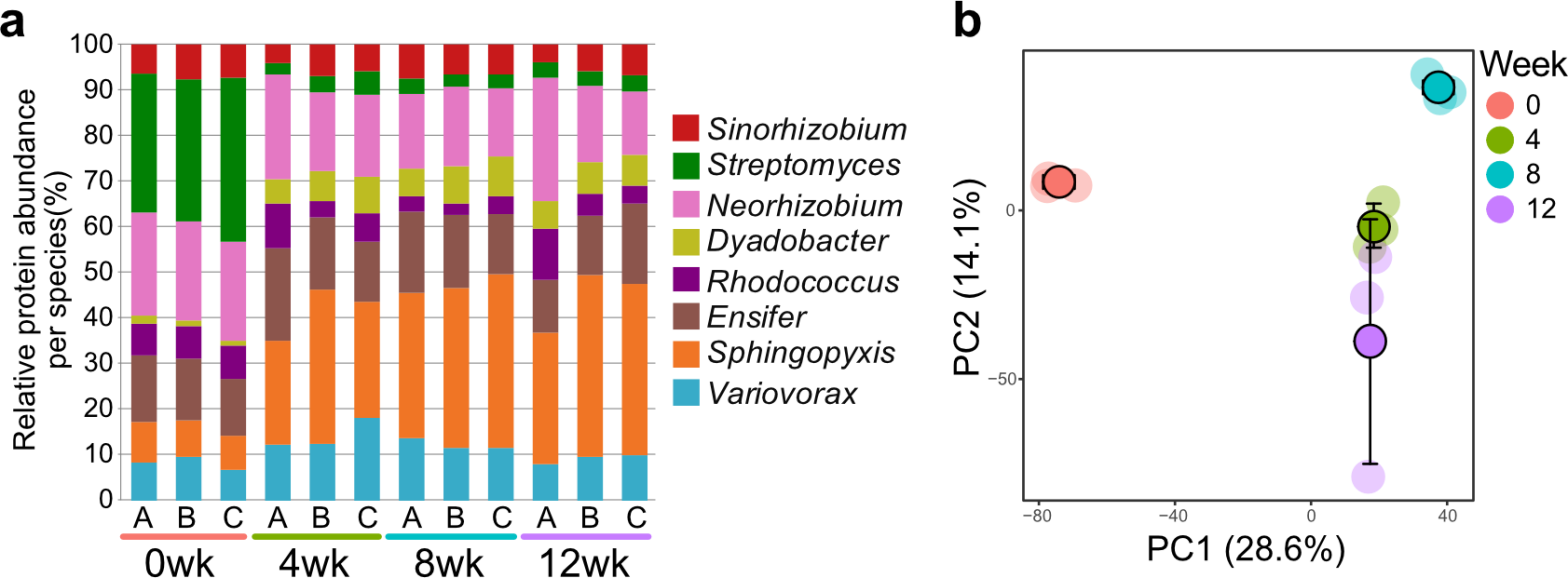
Shifts in MSC-2 protein types and abundances over 12 weeks of incubation with chitin in soil. (**a**) Relative protein abundances for individual MSC-2 species over 12 weeks of incubation: 0 weeks (red), 4 weeks (green), 8 weeks (blue), and 12 weeks (purple). Letters below columns correspond to individual replicates. (**b**) PC1 vs PC2 of the score plot of the PCA resulting from the proteomics data of all incubation time periods. Faded dots represent individual samples, and deep-colored dots with black outlines represent the average value (error bars ± SD) for each of the incubation periods on the plane defined by the PC1 vs PC2.

**Fig 4:**
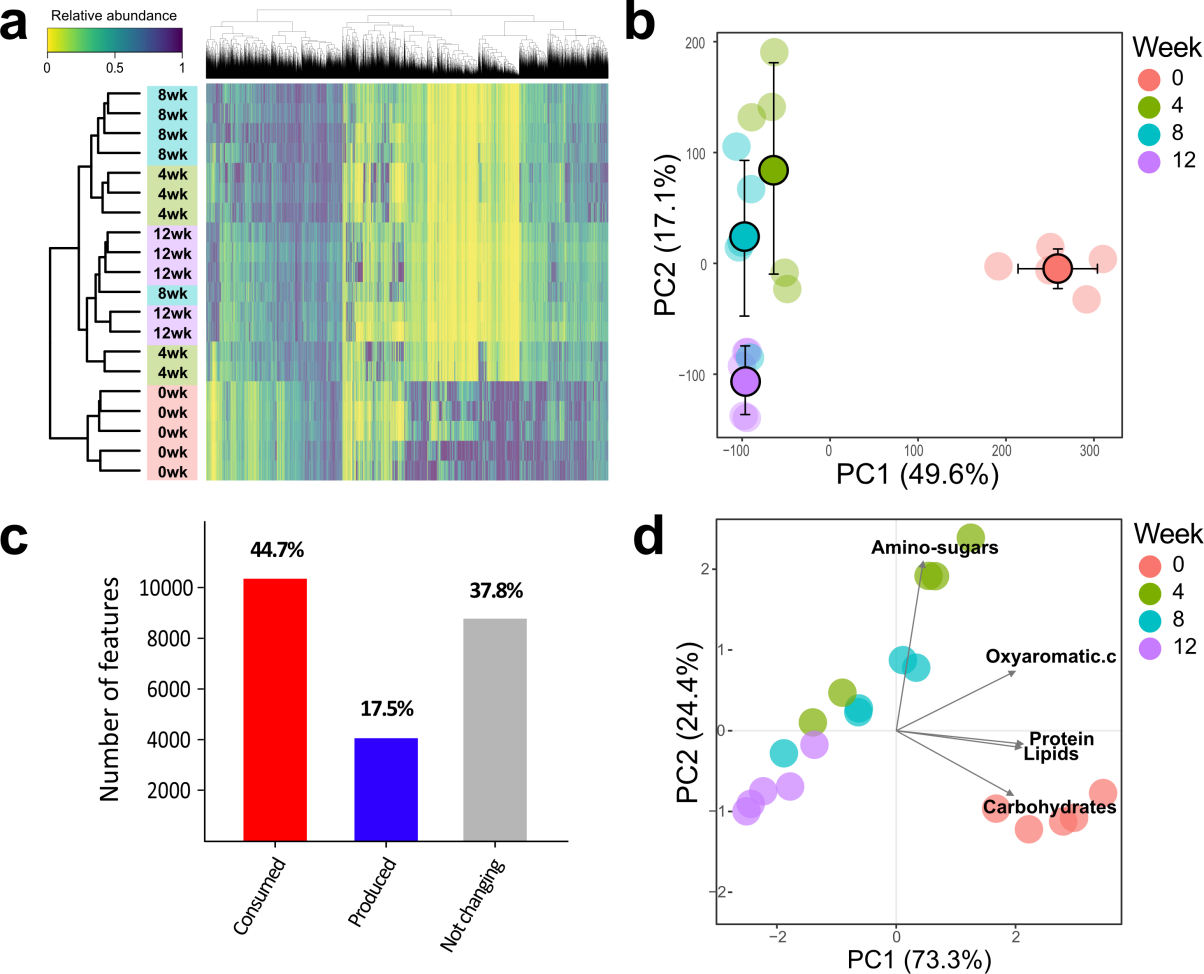
Shifts in metabolites over 12 weeks of incubation in soil. (**a**) Hierarchical clustering of both metabolomic features (top) and MSC-2-inoculated samples across incubation weeks (left) coupled to a heatmap with the relative abundance of the metabolomic features in each sample. The most abundant features are shown in dark blue, while low-abundance features are shown in yellow. Weeks of incubation: 0 weeks (red), 4 weeks (green), 8 weeks (blue), and 12 weeks (purple). (**b**) PC1 vs PC2 of the score plot of the PCA resulting from the metabolite fingerprints of all incubation weeks. Faded dots represent individual samples, and deep-colored dots with black outlines represent the average values (error bars ± SD) for each of the incubation weeks along the PC1 and PC2. (**c**) Relative number of metabolomic features with reduced (consumed) and increased (produced) relative abundances and not changing (*P*-value > 0.05) between 0 and 12 weeks. (**d**) PC1 vs PC2 of the PCA biplot resulting from a data set including the total abundances of features classified as amino-sugars, oxyaromatic compounds, protein, lipids, and carbohydrates.

The relative amount of RNA from each of the eight species was determined across the four time points (Fig. 2). The total alignment percentage of the RNA-seq data across all samples ranged from 91 to 95%. We include absolute amounts of RNA from each species in Fig. S6. In contrast to the amplicon data, *Rhodococcus* had the highest relative transcript abundance over the time course of the study (Fig. 2a). Like the species abundance data (Fig. 1), the largest changes in gene expressions occurred between 0 and 4 weeks (Fig. 2b; Table S6). For example, *Neorhizobium* transcripts had a relative abundance of ~20% at time 0 but decreased to ~3% at 4 weeks. This shift corresponded to a relative increase in *Dyadobacter* transcripts from ~3% at time 0 to ~15% at 4 weeks (Fig. 2a). The PCA plot of the samples also showed major shifts from week 0 to subsequent weeks with fewer changes at subsequent time points (Fig. 2b). The observation that RNA profiles do not change dramatically after the initial shift from 0 to 4 weeks contrasts with our 16S rRNA sequencing data (Fig. 1), as well as subsequent analyses of proteins and metabolites (Fig. 3 and 4), where moderate changes continued to take place throughout the experiment. We hypothesize that the rapid response of bacterial species to new stimuli at the RNA level, a response that is likely faster than changes in abundance or shifts in proteins or metabolites, is the main driver of this observation (14).

There were large differences between species with respect to their differentially expressed genes (DEGs) (Table S6). After normalizing for the size of the genome, *Rhodococcus, Variovorax,* and *Ensifer* had the highest percentage of DEGs, when comparing 0 to 4 weeks (55%, 41%, and 40% of genes were DEGs, respectively). However, these species showed some of the smallest changes in species abundance over time. In contrast, *Streptomyces and Dyadobacter* had the fewest DEGs (18% and 16%, respectively), although they showed the greatest changes in abundance over time—both initially increasing and then declining. When comparing 4 to 12 weeks, the number of DEGs for all members was far less than that seen at 0 to 4 weeks (Table S6), indicative of a decline in gene regulation as cells became more acclimated to their new soil environment and chitin substrates became depleted. Comparing 4 to 12 weeks of incubation, *Variovorax* and *Rhodococcus* had the highest percentage of DEGs (6.1% and 5%), while *Sinorhizobium* and *Streptomyces* had the lowest percentage of DEGs (0.12%, 7 genes and 0.53%, 38 genes, respectively).

### Metaproteomics of MSC-2 in soil during chitin decomposition

The metaproteome data were used to determine proteins involved in chitin decomposition that were produced by the eight MSC-2 species during the incubation (Table S7). At time 0, samples from soil inoculated with 10^8^ cells per gram of soil had a lower protein yield compared to the samples with 10^9^ cells per gram, reflecting the tenfold differences added in cell biomass. (Fig. S7). This corresponds to our DNA analysis above where soil inoculated with 10^8^ cells has less DNA at weeks 0, 4, and 8 compared to soil inoculated with 10^9^ cells per gram of soil (Fig. S2).

When focusing on the higher inoculum dose at the individual species level, *Streptomyces* had the highest relative amount of assigned proteins at week 0 (Fig. 3a), when it also had the highest relative abundance (Fig. 1). *Streptomyces* relative protein abundance subsequently declined between 0 and 4 weeks (11-fold), with corresponding increases in relative amounts of *Sphingopyxis* (fourfold) and *Dyadobacte*r (sixfold) proteins. There were also 1.5-fold decreases in relative protein abundances for *Neorhizobium* and *Variovorax* (Fig. 3a). Smaller shifts in relative protein abundances occurred between subsequent time points (Fig. 3). The PCA of the protein data set revealed that the 0 week time point was the most dissimilar and clustered separately from the other time points, which were separated along PC2 (Fig. 3b).

### Metabolite analyses in soil during chitin decomposition

There were significant differences in overall metabolite compositions between consecutive weeks. Similar to the other omics analyses, hierarchical clustering and PCA analyses of metabolite data showed the largest change between week 0 and consecutive weeks (Fig. 4a and b). Analyses of individual features between 0 and 12 weeks revealed that 44.7% of the features were reduced in abundance (consumed) over time, 17.5% were produced (bacterial metabolism products), and 37.8% did not undergo changes (*P*-value > 0.05) (Fig. 4c). The PCA with compound classes (Fig. 4d) showed an overall higher abundance of amino sugars at 4 weeks. This finding could be explained by the accumulation of the chitin monomer, N-acetylglucosamine, as chitin levels likely dropped over time due to metabolism, but the production of these molecules by other species, irrespective of chitin breakdown, cannot be fully discounted. The relative abundances of the other compound classes were highest at week 0 and declined over time (Fig. 4d; Fig. S8), indicative of consumption of the added chitin substrate and decomposition of cellular components from the added inoculum. Regarding individual metabolomic features, 26% of all detected features underwent significant changes (*P*-value < 0.05) between 0 and 4 weeks. This percentage decreased to 7.6% and 7.2% when comparing 4 to 8 weeks and 8 to 12 weeks, respectively (Table S8).

Principal component analysis (PCA) of metabolite fingerprints for all control and inoculated samples revealed that the control samples clustered together with the 0 week inoculated samples along principal component (PC) 1 (Fig. S9). One-way ANOVA on the score coordinates did not reveal significant clustering between control samples at different weeks along both PC1 and PC2 of the PCA as well as in a PCA performed solely with the controls (Fig. S9). Analysis performed with the metabolomic fingerprints of control samples of consecutive measured weeks (0 versus 4 weeks; 4 versus 8 weeks; 8 versus 12 weeks) showed no significant differences (Table S8). These results indicate that the composition of the natural soil organic matter, in terms of abundance and compound diversity, did not change across time in the absence of the inoculum. Therefore, metabolite differences between measured weeks in inoculated samples were driven only by the inoculum.

### Multi-omic analysis of the chitin decomposition pathway

#### Metatranscriptome data

**Fig 5:**
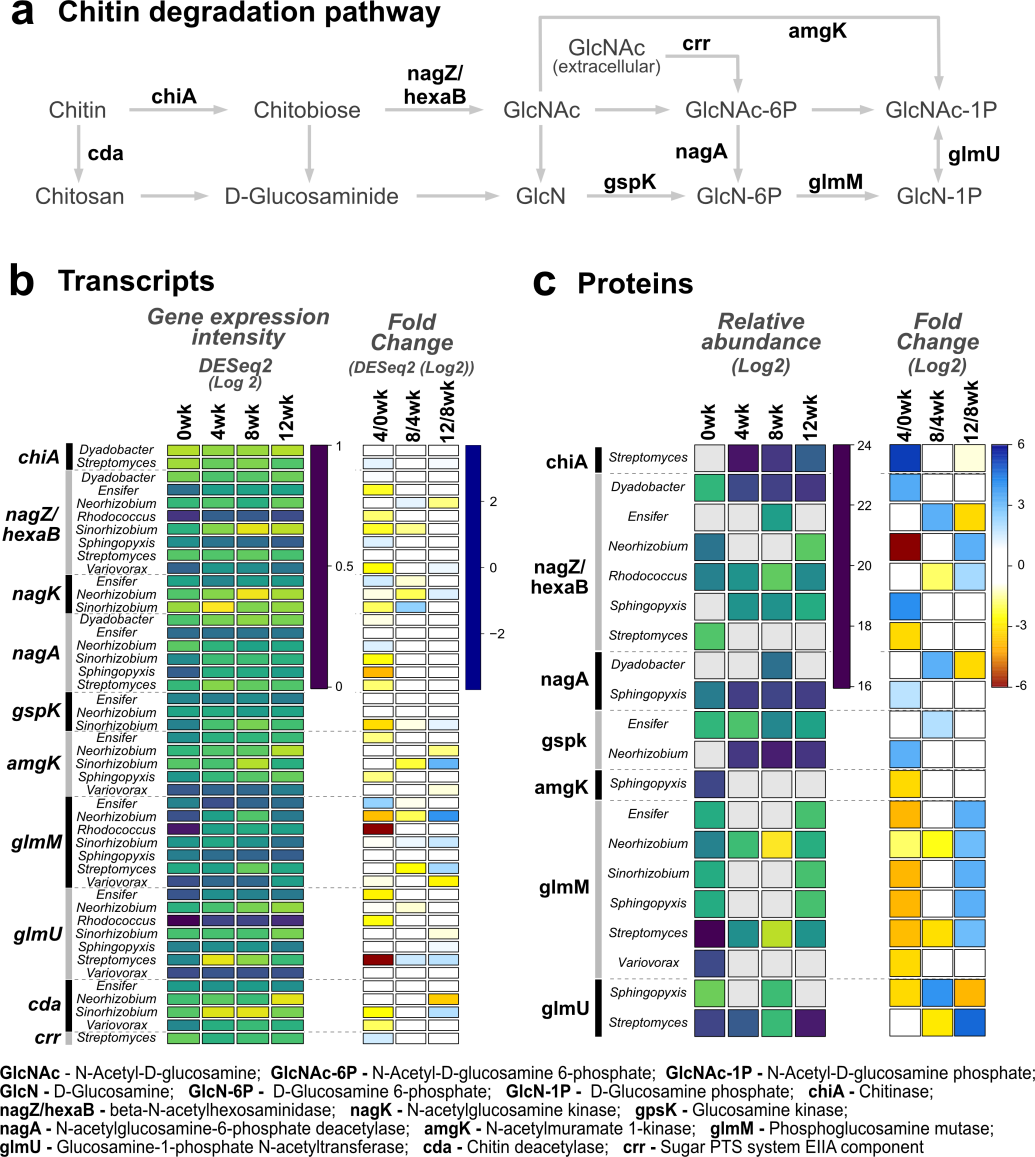
Gene expression and enzyme abundance of the chitin degradation pathway across incubation periods. (**a**) Chitin degradation pathway, compounds, and identified transcripts/enzymes are shown in dark gray and black, respectively. (**b**) Heatmaps for the normalized gene expression intensity DESeq2 (log2) and the log2 fold change of the DESeq2 data (right) between consecutive incubation periods. The highest expression level or fold change value is shown in dark purple or blue, respectively. Lower expression levels are shown in yellow and lower fold change values in orange/red. (**c**) Heatmaps for the enzymatic relative abundance (log2) (left) and their fold change (right) between consecutive incubation periods. Color schemes match that in panel b).

The RNA-sequence reads were normalized via RPKM to compare the expression by each species/gene at each time point (Fig. 5a). *Streptomyces* emerged as the initiator of chitin polymer degradation, followed by *Dyadobacter* (Fig. 5a). Specifically, both species presented upregulation of transcripts for extracellular chitinase enzymes in the GH18 family at 4 weeks compared to 0 weeks (RPKM fold change of 2.94 and 3.48, respectively). *Streptomyces* showed maximum expression at 12 weeks with a fold change of 3.98 with respect to 8 weeks (Fig. 5a). Subsequently, all eight species expressed *nagZ/hexaB* genes for the next step in the conversion of chitobiose to N-acetylglucosamine. Note that both *nagZ* and *hexaB* gene designations are used for the same enzymatic reaction, so we included both here. However, there were differences in expression from the species over time. For example, *Rhodococcus* expression of *nagZ/hexaB* declined after week 0, suggesting that it was downregulated. Transcription increased for *Sphingopyxis*, suggesting that it was upregulated, and it was constitutive for most other species.

The *nagK* gene, coding for an enzyme that converts N-acetylglucosamine to N-acetylglucosamine-6-P, was expressed by *Ensifer*, *Neorhizobium,* and *Sinorhizobium*. The same three species also expressed *gspK* that phosphorylates the deacetylated form (glucosamine). Six out of the eight species expressed *nagA* involved in deacetylation of N-acetylglucosamine-6-P to glucosamine-P. Additionally, several species expressed *amgK,* a sugar kinase that forms N-acetylglucosamine-1-P. All species, except for *Dyadobacter*, expressed *glmM*, encoding a phosphoglucosamine mutase that catalyzes the formation of glucosamine-1-phosphate from glucosamine-6-phosphate, an essential step in the pathway for UDP-N-acetylglucosamine biosynthesis in bacteria. Finally, all species, except *Dyadobacter,* expressed glmU*,* an enzyme that catalyzes the last two sequential reactions in the biosynthetic pathway for UDP-N-acetylglucosamine by using acetyl coA and alpha-D-glucosamine-1-P as substrates. It uses acetyl CoA and alpha-D-glucosamine-1-P as substrates. In addition, *Ensifer, Neorhizobium, Sinorhizobium,* and *Variovorax* expressed the *cda* gene that encodes a chitin deacetylase that removes acetyl groups from chitin to form chitosan and release acetic acid. *Streptomyces* also expressed the *crr* gene that encodes a sugar transporter that could be involved in the transport of glucosamine over the cell membrane, during which process the sugar is phosphorylated. Normalized log2-transformed expression levels for all genes and all species are found in Data set S1 at https://data.pnnl.gov/group/nodes/dataset/34237.

#### Metaproteomics data

*Streptomyces* chitinase proteins were detected for the first step of the chitin degradation pathway, but in contrast to the metatranscriptomes, no *Dyadobacter* proteins were detected (Fig. 5b). For *Streptomyces*, two ChiA proteins were identified from the genome (CDS.2626 and CDS.6268; both annotated as GH18 chitosanases). These were not detected at the 0 week time point but were found at later weeks, suggesting that their production was upregulated under conditions of chitin accessibility. However, at 4 weeks, G1_CDS.2626 was the more abundant ChiA protein, which then steadily decreased over the subsequent weeks. By contrast, G1_CDS.6268 was only 50% and 33% as abundant as CDS.2626 at 4 weeks and 12 weeks, respectively, with the highest abundance at 8 weeks. These data suggest that while these two enzymes may work in tandem, as chitin availability decreases, CDS.2626 is likely the enzyme that contributes more to chitin degradation than CDS.6268.

The metaproteome data (see Data set S2 at https://data.pnnl.gov/group/nodes/dataset/34237.) supported the metatranscriptome data, suggesting that chitobiose was degraded into N-acetylglucosamine monomers by NagZ/HexaB enzymes. However, not all species that expressed these genes produced detectable amounts of the protein. In addition, different species produced these proteins at different times. *Dyadobacter*, *Neorhizobium, Rhodococcus, and Streptomyces* produced relatively equal amounts of NagZ/HexaB proteins (G5_CDS.1705) at 0 weeks. Subsequently, NagZ/HexaB enzyme production increased primarily in *Ensifer* (>2 fold) between 4 and 8 weeks. Between 8 and 12 weeks, NagZ/HexaB decreased ~2 fold in *Sphingopyxis* and *Dyadobacter. Streptomyces* NagZ/HexaB proteins were not detected after the first sampling point (0 weeks), and *Neorhizobium* proteins were detected only at 0 weeks and 12 weeks of incubation. *Ensifer* NagZ/HexaB was only observed at the 8 week time point.

In contrast to the metatranscriptome data, no peptides were detected for NagK. However, GspK proteins were produced by *Ensifer* and *Neorhizobium*; two of the same three strains for which we observed gene expression, with the exception of *Sinorhizobium* (Fig. 5). The AmgK protein, which catalyzes phosphorylation of N-acetylglucosamine to N-acetylglucosamine-1-P, was only detected for *Sphingopyxis* and only on the week 0 sampling point, although five of the eight strains in MSC-2 transcribed the gene. Additionally, *Sphingopyxis* was the primary producer of detectable proteins from NagA, which catalyzes deacetylation of N-acetylglucosamine-6-P to glucosamine-6-P. The amount of NagA protein increased ~4 fold between 0 and 4 weeks and then held was maintained at the same level for the remaining incubation period over 12 weeks. *Dyadobacter* also had detectable NagA at the 8 week time point (Fig. 5b).

Both transcripts and proteins were detected for phosphoglucosamine mutase, which catalyzes the formation of glucosamine-1-P from glucosamine-6-P (Fig. 5). This enzyme can directly utilize the product of GspK (glucosamine-6-P) and initiates the biosynthesis of UDP-N-acetylglucosamine. The GlmM protein was produced by six out of the eight species, the exceptions being *Dyadobacter* and *Rhodococcus*. These data were similar to the metatranscriptome data, with the exception of transcripts detected for *Rhodococcus* at the beginning of the experiment. Subsequently, GlmU proteins, responsible for subsequent steps in UDP-N-acetylglucosamine biosynthesis, were only detected for *Sphingopyxis* at 0 and 8 weeks and for *Streptomyces* at all time points (Fig. 5b). In contrast to the metatranscriptome data, no proteins were detected for the chitin deacetylase (encoded by *cda* gene) or sugar transporter (encoded by *crr* gene) by *Streptomyces*. This could be explained by post-transcriptional modifications (15) or by differences in relative peptide abundances associated with each protein and with each strain, from which protein values can be found in Data set S3 (https://data.pnnl.gov/group/nodes/dataset/34237).

**Fig 6:**
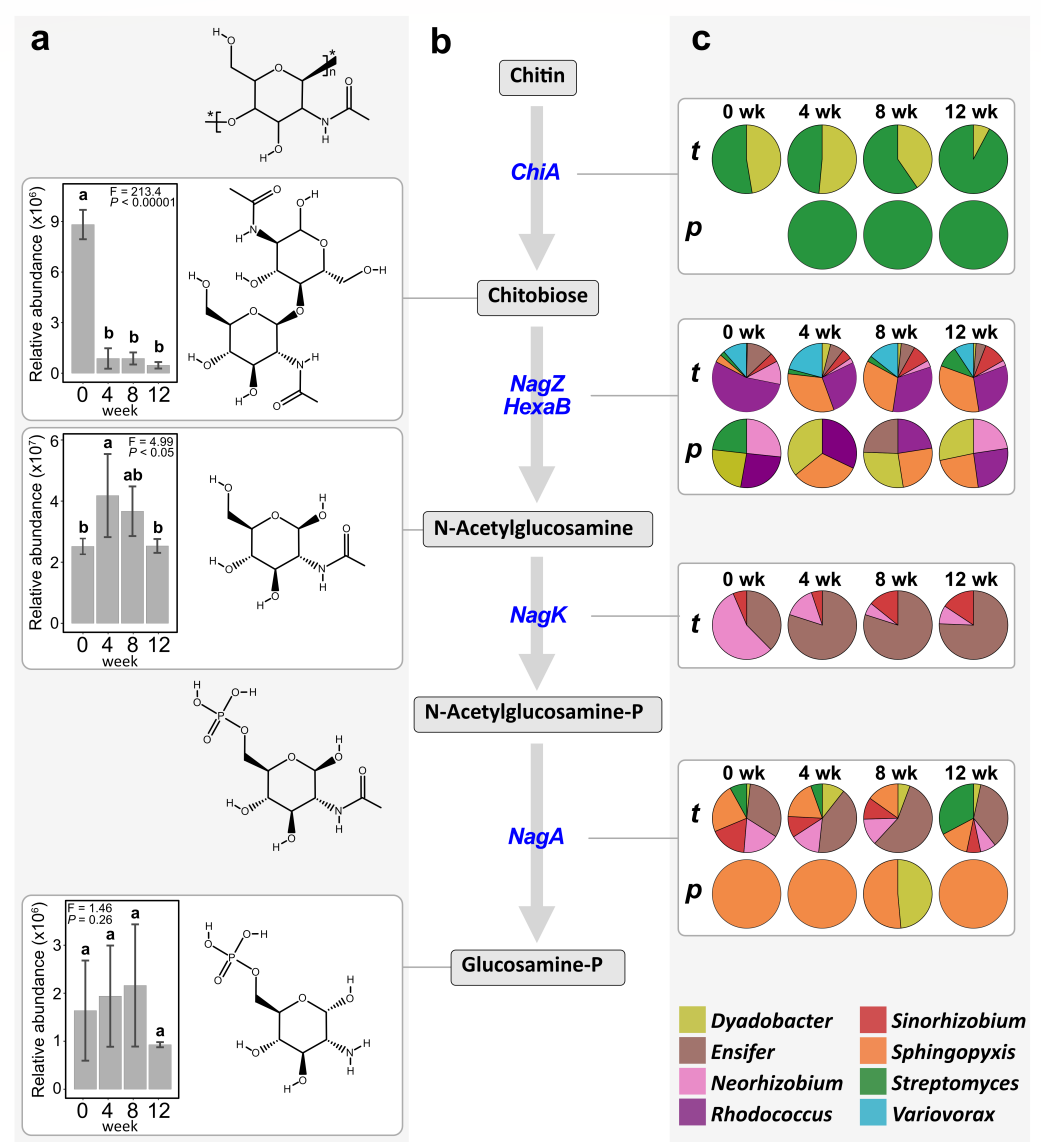
Chitin degradation pathway by MSC-2. One-way ANOVAs of relative abundances of identified metabolites along the chitin degradation pathway (**a**). The bar graphs denote statistically significant differences (*P*-value < 0.05) after HSD Tukey post hoc test. Fisher F and *P* values are indicated. Bars with different letters represent significant differences from each other. Central chitin degradation pathway according to KEGG map00520 (**b**). Pie charts indicating the relative abundances of transcripts (*t*) and enzymes (*p*) upregulated corresponding to individual MSC-2 microbial species for each enzyme of the chitin degradation pathway (**c**). Different microbe species are shown with different colors. Incubation times are represented above the pie charts as 0 wk, 4 wk, 8 wk, and 12 wk for 0, 4, 8, and 12 weeks, respectively.

Figure 6 shows the relative contributions of proteins and transcripts identified from each species in the chitin degradation pathway. It is important to note that RNA-seq is a more sensitive analytical method compared to proteomics, which is limited to what a mass spectrometer can detect from typically the most abundant proteins, typically 60%–80% of all proteins (16). Transcripts observed from each of the species studied here might also have produced proteins that are below the limits of detection of the mass spectrometer. The proteins detected likely represent the most abundant from each enzyme class in the pathway. The protein data, while limited in sensitivity compared to the transcriptomics, are complementary and can validate which proteins of each enzyme in the pathway are produced in the highest relative quantities, indicating which proteins are most likely involved in a particular pathway.

#### Metabolite data

Several metabolites of chitin decomposition were detected, including chitobiose (KEGG ID: C01674), N-acetylglucosamine (KEGG ID: C00140), and glucosamine-P (KEGG ID: C00352) (Fig. 6). Chitobiose was highest at 0 weeks and reduced at 4 weeks as it was decomposed, with no changes detected during following weeks. N-acetylglucosamine accumulated at 4 weeks and was consumed during the following weeks. The phosphorylated form, N-acetylglucosamine-P, was not detected and was presumed to be a transient intermediate. Glucosamine-P was detected and tended to be highest at 8 weeks, although it did not significantly change over the incubation period. The metabolite data complemented the expression data and allowed us to derive the pathway for chitin metabolism by MSC-2 (Fig. 6).

## DISCUSSION

This study reveals how different members of a soil microbial consortium express different chitin metabolism enzymes at different stages of chitin breakdown in a soil matrix. Because we used a consortium with well-characterized species, we could assign specific steps in chitin degradation to specific microbes. Previously, MSC-2 species dynamics during chitin decomposition were studied under well-mixed liquid conditions (10, 11). The soil environment is, however, very different from a liquid setting. For example, soil microorganisms are subjected to physical constraints and a heterogeneous mixture of soil particles and microhabitats (17, 18). Because of this, and other differences between the two experiments, such as different time scales, we refrain from making direct comparisons between the two experimental conditions as this was not the scope of the current study. Here, to approximate natural conditions, we inoculated MSC-2 into the same source soil that the species were originally isolated from and compared species to identify specific roles in chitin degradation in soil across time. In addition, because the soil was sterilized, there was no competition from indigenous microorganisms, which would otherwise occur.

Analysis of the multi-omics data showed that MSC-2 members act in a successive manner through division of labor to drive chitin decomposition in soil (Fig. 6). Because the individual strains were naturally enriched together during growth on chitin, this finding supports our initial hypothesis that selective enrichment would result in a consortium with metabolic interdependencies. *Streptomyces* emerged as a key player that carried out the initial step of conversion of chitin to chitobiose, with some contribution from *Dyadobacter*. The importance of *Streptomyces* for the initiation of chitin decomposition by MSC-2 was also suggested in liquid growth studies. Expression of genes for chitinase degradation has been shown to be upregulated by chitin availability (chitin levels lead to induced expression of chitinase genes) in *Streptomyces coelicolor* A3(2) (19). This is likely occurring with the *Streptomyces* sp001905665 strain 001 in our study, based on its significant increase in chitinase transcripts and proteins following exposure to chitin. However, the degradation product, N-acetylglucosamine, has been shown to downregulate the expression of chitinase genes in *Streptomyces* (20). Therefore, as N-acetylglucosamine levels increase, *Streptomyces* may be repressed in its ability to degrade chitin. Interestingly, although *Streptomyces* decreased in relative abundance over time, the chitinase enzyme(s) persisted, suggesting that the proteins are relatively robust or that continued translation from expressed mRNAs for chitinases was maintained. The drop in *Streptomyces* relative abundance also tracks with our metabolomics analysis showing increases in chitobiose early in the experiment, suggesting that much of the chitin breakdown takes place in the first 4 weeks and that subsequent metabolic processes may be driven by chitin breakdown products. However, we note that the accumulation of metabolic intermediates is the net result of production and consumption/degradation. Hence, a transient peak in a metabolite does not mean they are not produced earlier or later, just that the production dominates over consumption at that time point. These data exemplify the value of multi-omics analyses because interpretations from 16S rRNA analyses alone would have downplayed the relative importance of *Streptomyces* to long-term functioning of the community. This is also an example of a primary contribution by one member of the consortium that enables other members to grow on chitin breakdown products.

Conversion of chitobiose to N-acetylglucosamine by NagZ/HexaB was carried out by several consortium members (Fig. 6). However, only three species (*Ensifer, Neorhizobium*, and *Sinorhizobium*) transcribed the *nagK* gene responsible for phosphorylation of N-acetylglucosamine (Fig. 5). Several species transcribed the *nagA* gene, with the highest expression across time points for *Ensifer*. However, there were discrepancies between data sets, with the protein data showing the highest yields for the *Sphingopyxis* NagA enzyme across all time points and at 8 weeks only for *Dyadobacter* (Fig. 5). Finally, 7 out of 8 members of MSC-2 (with the exception of *Dyadobacter*) carried out the following UDP-N-acetylglucosamine biosynthesis steps from glucosamine-6-P: conversion to glucosamine-1-P by GlmM and production of UDP-N-acetylglucosamine by GlmU. UDP-N-acetylglucosamine is a precursor for cell-wall biosynthesis (21).

We note that alternate pathways are possible, and they are not mutually exclusive. For example, the AmgK enzyme phosphorylates N-acetylglucosamine in the first position to produce N-acetylglucosamine-1-P, and this protein was produced by *Sphingopyxis*. In addition, chitin can be directly deacetylated to produce chitosan (7, 8), as suggested in the metatranscriptome data by expression of the *cda* gene (encoding a chitin deacetylase) by four of the MSC-2 species. However, the corresponding protein was not detected. Additionally, *Streptomyces* transcribed a sugar transporter encoded by the *crr* gene, suggesting that this is an alternative route for uptake of chitin sugar monomers into the cells.

The results of the Pearson correlations revealed positive and negative co-abundances, and thus possible interactions, between the different MSC-2 species. Species that were positively correlated likely benefited from metabolic sharing or other beneficial interactions (mutualistic relationships). The results (Fig. 1) show two groups with species that are positively correlated: *Ensifer Variovorax, Dyadobacter,* and *Sphinopyxis* (Group 1) and *Rhodococcus, Neorhizobium, Streptomyces,* and *Sinorhizobium* (Group 2). Another explanation for the positive correlations is that they simply responded to similar environmental cues. However, Groups 1 and 2 were negatively correlated with each other, suggesting that the member species were antagonistic or competitive during growth on chitin. An alternate explanation could be that they are carrying out different steps in the degradation pathway, and thus their species abundances reflect the specific chitin metabolites available for their specific enzymatic capabilities. For example, by examination of the degradation pathway in Fig. 6, and expressed genes and proteins in Fig. 3 and 5, respectively, one explanation could be that initial conversion of chitin to chitobiose was a competition between *Streptomyces* and *Dyadobacter*, with *Streptomyces* (a member of Group 2) showing higher amounts of the protein. However, the breakdown of chitobiose to the N-acetylglucosamine monomer involved activities by members of both Groups 1 and 2, although *Rhodococcus* (Group 2) dominated at the protein level. Similarly, *nagK* transcripts were produced by members of both groups, although expression levels varied, shifting from a dominance by *Neorhizobium* (Group 2) at week 0 to *Encifer* (Group 1) at subsequent time points. *Sphingopyxis* (Group 1) dominated protein expression of NagA at all time points, except at 8 weeks when *Dyadobacter* (Group 2) produced roughly equal amounts of the protein. We note that other aspects of the environment may select for certain species in tandem that have little to do with their direct interactions.

There are several practical implications of this study. Because chitin is present in the cell walls of most soil fungi and several soil fungi are pathogenic, a chitin-degrading consortium, such as MSC-2, has the potential for biocontrol of soil fungal pathogens (22–24). In addition, this study demonstrates the ability to define key, testable hypotheses regarding the basic mechanisms underlying soil organic carbon decomposition by a naturally derived consortium of microorganisms. As such, the MSC-2 consortium serves as a valuable model system for testing specific hypotheses related to soil microbial ecology that have been difficult to test using highly complex and diverse natural soil systems. To date, there are only a limited number of well-characterized soil consortia (25, 26), and most of those have fewer members than MSC-2. By increasing the number of species that interact to carry out a specific function (i.e., chitin decomposition), it is possible to increase the complexity of the underlying models that can be leveraged for other microbial systems as well.

## MATERIALS AND METHODS

### Growth of MSC-2 organisms

We used a previously developed model soil consortium (Model Soil Consortium-2 [MSC-2]) (10) to monitor decomposition of chitin in soil. MSC-2 consists of eight members: *Streptomyces* sp001905665 strain 001, *Neorhizobium tomejilense* strain 005, *Dyadobacter* sp. strain 007, *Sphingopyxis* sp. strain 008, *Ensifer adhaerens* strain 011, *Variovorax beijingensis* strain 012*, Sinorhizobium meliloti* strain 014, and *Rhodococcus* sp003130705 strain 016. Each of the strains was plated on an R2A agar (Sigma-Aldrich) plate from a glycerol stock stored at −80°C and incubated for 1 week at 20°C. Resulting single colonies were used to inoculate individual 5 mL cultures in R2A. The 5 mL cultures of each strain were inoculated separately into 100 mL R2A and incubated overnight at 20°C with shaking at 180 rpm. Finally, those 100 mL cultures were used to inoculate 500 mL cultures of R2A, starting at an O.D. of 0.1, and incubated at 20°C with shaking at 180 rpm. Strains were spun down and washed with fresh R2A twice between inoculations. The starting incubation times were calculated so that each strain would reach the exponential phase (O.D. ~ 0.5) at approximately the same time on the following day. The biomass was collected by centrifugation for 5 min at 5,000 × *g*. Growth curves were used to determine the volume needed to establish a given cell concentration for each strain in soil (Table S1). The volume of the eight strains shown in Table S1 was collected by centrifugation (5 min, 5,000 × *g*) and resuspended in 10 mL of M9 medium (27) containing 50 µg/mL colloidal chitin from shrimp shells (Sigma). *Streptomyces* and *Rhodococcus* strains tend to clump, making CFU to O.D. comparisons difficult. Therefore, for each of these two strains, the complete 500 mL culture was used for incubation. The cultures were incubated overnight at 4 ° C to pre-adapt them for inoculation into soil containing chitin. Subsequently, 6 mL of each strain were combined into one tube to generate 48 mL of an MSC-2 master mix containing all eight strains at approximately equal cell densities. Prior work (Table S1) showed that adding 2 mL of this master mix to 20 grams of soil would lead to a cell concentration of 10^9^ cells per gram of soil.

### MSC-2 incubation in a soil matrix

Surface soil (upper 20 cm) was collected at the Washington State University Irrigated Agricultural Research and Extension Center (IAREC) in Prosser, WA, USA. A 550 g portion of the soil was autoclaved (90 min, Gravity Cycle). Following autoclaving, 75 mL of sterile Milli-Q water was added to the soil and mixed to establish a soil moisture content of 15% (wt/vol). The moistened soil was incubated for 2 days at room temperature before being re-autoclaved (90 min, Gravity Cycle). After this second round of autoclaving, 50 mL of sterile Milli-Q water was added to the soil to establish a soil moisture content of 10% (wt/vol), along with dry chitin powder to a final concentration of 500 ppm (0.5 mg chitin/g soil). The moistened soil was mixed with a sterile spatula and incubated in 2 L glass beakers for 24 h at room temperature in a biosafety cabinet.

Twenty-gram portions of pre-incubated sterile soil containing chitin were added to each of 52 50 mL conical tubes (Olympus Plastics, cat.# 28-106). The tubes were divided into three sets: (i) a high inoculation dose of 10^9^ cells of MSC-2 per gram of soil, (ii) a low inoculation dose of 10^8^ cells of MSC-2 per gram of soil, and (iii) uninoculated controls. For each of the inoculated samples, there were 20 tubes total with five biological replicates per each of four time points: 0, 4, 8, and 12 weeks. The uninoculated controls had three biological replicates per time point. For the samples inoculated with the higher inoculum dose, 2 mL of the MSC-2 master mix was added to each of the 20 gram sterile soil samples in the tubes; corresponding to 10^9^ viable cells (equally distributed among the eight MSC-2 strains) per gram of soil (Table S1). For the lower inoculum dose, 4.5 mL of the MSC-2 master mix was diluted 1:10 with M9 medium containing 50 µg/mL chitin, and 2 mL of the diluted master mix was added to each of the tubes; corresponding to 10^8^ viable cells (equally distributed among the eight MSC-2 strains) per gram of soil (Table S1). As noted above, 10^9^ samples were prepared in media containing 50 µg/mL chitin. Thus, both inoculum doses, 10^9^ and 10^8^, started with the same amount of chitin. Finally, 2 mL of sterile M9 containing 50 µg/mL chitin was added to each of the control tubes. The inoculated soils were separately mixed with a sterilized spatula. These experiments form the basis for a future study that aims to determine community dynamics under low gravity conditions on the International Space Station (ISS). To slow metabolism prior to incubation on the ISS, it was necessary to store inoculated samples at 4°C to allow time for transport to the ISS. Therefore, we included a 5 day preincubation at 4°C in this study as well.

Following this 5-day pre-incubation period, “0 week” samples were collected and stored at −80°C. The remaining samples were covered with foil to prevent inadvertent light exposure and incubated at 20°C. Sample tubes were subsequently removed from incubation after 4, 8, and 12 weeks of incubation and stored at −80°C. Time points were also chosen to match the needs of our subsequent space incubation experiments. At the conclusion of the complete experiment, all samples were processed for DNA, RNA, protein, and metabolite extraction as described below and shown in Fig. S1.

### Extraction and analysis of DNA and RNA from soil

DNA was extracted from 0.5 g aliquots of soil using the Quick-DNA Fecal/Soil Microbe Microprep kit (ZYMO Research, Irvine, CA). The DNA yields at each time point were calculated using a Qubit (Thermo Fisher, Waltham, MA). The DNA was sequenced using an Illumina MiSeq (Illumina, San Diego, CA) using 16S primers that target the V4 hypervariable region of the 16S small-subunit (SSU) rRNA gene using the V4 forward primer (515F) and V4 reverse primer (806R) and the MiSeq Reagent Kits v2. Reads were processed using QIIME2 (v2021.4). Within the QIIME2 environment, DADA2 (q2-dada2) under default settings was used to denoise the reads and cluster amplicon sequence variants (ASVs), and the SILVA database (v138) was used for taxonomy assignment (q2-feature-classifier).

RNA was extracted from 2 g portions of soil using the Quick-RNA Fecal/Soil Microbe Microprep kit (ZYMO Research, Irvine, CA). RNA integrity was confirmed on an Agilent Bioanalyzer (Agilent Technologies, Santa Clara, CA) with all RNA Integrity Numbers (RINs) above 7.0. RNA sequencing was carried out using Illumina technology by Genewiz (South Plainfield, NJ), with paired-end reads of 300 bp in length. The rRNA depletion sequencing library was prepared by using three probes from the QIAGEN FastSelect rRNA 5S/16S/23S Kit (Qiagen, Hilden, Germany). RNA sequencing library preparation used the NEBNext Ultra II RNA Library Preparation Kit for Illumina by following the manufacturer’s recommendations (NEB, Ipswich, MA, USA). Briefly, enriched RNAs are fragmented for 15 min at 94°C. First-strand and second-strand cDNA were subsequently synthesized. cDNA fragments were end-repaired and adenylated at 3’ ends, and universal adapters were ligated to cDNA fragments, followed by index addition and library enrichment with limited cycle PCR, again according to the manufacturer’s instructions. Sequencing libraries were validated using the Agilent TapeStation 4200 (Agilent Technologies, Palo Alto, CA, USA) and quantified using Qubit 3.0 Fluorometer (ThermoFisher Scientific, Waltham, MA, USA) as well as by quantitative PCR (KAPA Biosystems, Wilmington, MA, USA) using primers against the Illumina adapter regions. The reads were aligned to a concatenated metagenome of each of the eight species’ individual genomes using the Burrows-Wheeler Aligner (28). Resulting SAM files were then converted to raw count files using HTSeq (29). Transcripts were normalized using the DESeq2 package within the statistics program R, which was also used to identify differentially expressed genes (DEGs) that were defined as genes with an adjusted *P*-value of ≤0.05 and absolute value fold change value of >2 comparing any two time points (30). Normalization was applied to each species separately so that abundance changes would not be misinterpreted as gene expression changes. PCA plots of metatranscriptomic data were done using these assignments to genes, not merely to species.

### MPLEX metabolite and protein extraction

Briefly, for each sample, 6 g of lyophilized soil was weighed into 50 mL centrifuge USP Class VI chemical-resistant PP tubes (Olympus Plastics 28-106, Center Valley, PA), and 6 mL of chloroform-washed stainless steel and of garnet beads (1:1) were added. Using a serological pipette, 12 mL of ice-cold 2:1 CHCl_3_:MeOH (vol/vol) and 4 mL of ice-cold ultrapure (18.2 megaohms-cm) water were added to each tube, and the tubes were placed on ice. The samples were vortexed horizontally at 4°C for 10 min and placed at −80°C for 15 min to cool them down. Samples, on ice, were sonicated for 30 s using a probe sonicator at 60% amplitude, and the samples were again placed at −80°C for 15 min. All samples were vortexed and sonicated one more time as described above. The tubes were centrifuged at 4°C for 5 min at 4,000 × *g*. The centrifugation step separated the emulsion into four distinct layers. The uppermost layer was an MeOH:H_2_O layer containing polar/hydrophilic metabolites. The second layer from the top was a precipitated protein disk layer that was extracted for proteomics analysis. The third layer contained chloroform with nonpolar/hydrophobic lipids. The residual soil remained at the bottom of the tube as a fourth layer. The samples were placed on ice, and 4.5 mL of the upper metabolite layer was collected from each tube and transferred into the corresponding tube of a pre-labeled set of 15 mL centrifuge USP Class VI chemical-resistant PP tubes (Olympus Plastics 28-101, Center Valley, PA).

Metabolite extracts (uppermost layer) were dried overnight (12 h) using a nitrogen evaporator and subsequently resuspended with 0.4 mL of 1:1 MeOH:H_2_O (vol/vol). Concentrated extracts were placed into a labeled set of 2 mL centrifuge tubes and centrifuged at 13,000 *× g* for 5 min at room temperature (24°C). Supernatants (150 µL) were collected and transferred into clean HPLC vials. Extracts were stored at −80°C until liquid chromatography tandem mass spectrometry (LC-MS/MS) analyses.

### Protein analyses

#### Protein digestion and sample cleanup/desalting

Protein pellets obtained from the MPLEx extraction were dried down (120 min) with a speed vac to remove residual methanol and chloroform. Dried pellets were re-wetted in 1.4 mL protein solubilization solution (7 M urea, 2 M thiourea, 4% CHAPS in nanopure water) and incubated at 4°C overnight. The samples were then incubated at 60°C for 1 h followed, by the addition of 2 mL of 100 mM ammonium bicarbonate in nanopure water, pH 7.8. The soil was vortexed for 5 min and then centrifuged at 3,000 × *g* for 6 min to sediment the soil particles. Then, 10 µL of the solution above the soil pellet was used for a Coomassie Plus (Thermo Scientific, San Jose, CA) assay to obtain a cursory estimate of the protein concentration and total protein mass according to the manufacturer’s instructions, using a bovine serum albumin standard. As protein or protein/peptide-like compounds can remain in soil even after autoclaving, average protein mass values between the control and inoculated soils provide an estimate of protein contributions made by the inoculum in each sample. Then, 10 mL of 100 mM ammonium bicarbonate in ultrapure (18.2 megaohm-cm) water (pH 7.8) was added to each sample along with 200 µL of 0.5 M neutralized Bond Breaker TCEP (Thermo Scientific, San Jose, CA) and 20 µL of 1 M CaCl_2_ solution. The samples were gently mixed, and trypsin (Affymatrix/USB, Santa Clara, CA) was added at a 1:20, protease:sample (wt/wt) ratio. The samples were incubated with trypsin for 10 h at 37°C. Then, 200 µL of 1 M chloroacetamide in ultrapure (18.2 megaohm-cm) water was added to alkylate reduced cysteines, and the samples were incubated in the dark for 30 min at room temperature.

#### Peptide clean-up/desalting and humic acid/fulvic acid removal

A strong cation exchange (SCX) solid-phase extraction (SPE) was used as an initial clean-up step to remove the bulk humic/fulvic acids as well as other particulate matter. To condition 3 mL volume size Agilent Bond Elut SCX SPE cartridges (500 mg, 120 µm), 3 mL of MeOH was flushed through the cartridges, followed by 14 mL of 10 mM ammonium formate in 25% ACN (pH 3.0). Each sample was centrifuged at 5,000 × *g* for 5 min to separate and remove the soil from the supernatant. Supernatants were transferred to new 15 mL tubes, and formic acid was added (~280 µl) to 14 mL of each post-digestion sample until the pH was ~2.5–3.0, as verified by pH paper. After formic acid addition, samples were gently mixed using slow up-and-down pipetting until off-gassing subsided. Then, tubes were quickly inverted a few times and decapped to allow further off-gassing. Each sample was spun-down at 7197 × *g* for 10 min. Approximately 4–5 mL of each supernatant was then slowly added to the SPE cartridge no faster than 1 mL/min. Retained peptides and humic acids were washed with 21 mL of 10 mM ammonium formate in 25% ACN, pH 3.0. All of the liquid was expelled from the SPE cartridge, and peptides and bound humic acids were eluted with 1.5 mL of 80:15:5 MeOH:H_2_O:NH_4_OH into a clean 2 mL microcentrifuge tube. Samples were dried in a speed-vac at room temperature until dry (~ 3 h).

Dried samples were resuspended in 200 µL of 1M NaCl in 1% (vol/vol) formic acid (pH ~3). Using the method proposed by Qian et al. (31), humic acids and residual CHAPS were filtered away from the peptides by passing the sample through a 10 kDa cutoff spin column filter (NanoSep Omega (modified polyethersulfone membrane), Pall, Port Washington, NY). Samples in the spin column filter were centrifuged at 4,500 × *g* for 30 min. Liquid that passed through the filter was collected and placed in a 1.5 mL microfuge tube. The addition of 200 µL of 1M NaCl in 1% (vol/vol) formic acid (pH ~3), followed by centrifugation and concatenation of all flowthroughs, was performed twice more. Concatenated flow-throughs were dried in a speed vac and resuspended in 200 µL of nanopure water using a sonicating water bath (~1 min) to aid in resolubilization of peptides.

#### Off-line HPLC fractionation of peptides

Using the HPLC method previously reported (Hixson et al., 2021), high pH reverse-phase HPLC fractionation was performed as a first dimension separation on each sample. Specifically, a Waters XBridge C18 column (250 mm × 4.6 mm i.d., 5 µm particle size) and a guard column (4.6 mm × 20 mm) were used with an Agilent 1200 HPLC System. Each sample loaded onto the C18 column was washed for 15 min with solvent A (10 mM ammonium formate, adjusted to pH 10 with ammonium hydroxide [14.8 M]). The gradient started with a linear increase of 0% solvent B (10 mM ammonium formate, pH 10, acetonitrile:ultrapure water [90:10 vol/vol]) to 5% solvent B over 10 min, 45% solvent B over 65 min, and then 100% solvent B over 15 min. Solvent B was held at 100% for 10 min and then was changed to 100% solvent A, this being held for 20 min to recondition the column. The flow rate was 0.5 mL min^−1^. A total of 48 fractions were collected for each sample into a 96 well plate. The fractions were then combined into six using the concatenation strategy previously reported (32). Peptide fractions were dried down and resuspended in ultrapure water at a concentration of 2 µg/uL for mass spectrometry analysis using a Q-Exactive Orbitrap MS (ThermoScientific, San Jose, CA).

#### LC-MS analysis of peptides

HPLC and mass spectrometry parameters were set as reported previously (33). In brief, the LC was configured to load the sample first on an SPE column, followed by separation on an analytical column. Analytical columns were made in-house by slurry packing 3 µm Jupiter C18 stationary phase (Phenomenex) into a 70-cm-long, 360 µm OD × 75 µm ID fused silica capillary tubing (Polymicro Technologies). Samples were loaded on the SPE column via a 5 µL sample loop for 30 min at a flow rate of 3 µL/min and then separated by the analytical column using a 60 min gradient from 99% mobile phase A (MP-A) to 5% MP-A at a flow rate of 0.3 µL/min. MS analysis was started 15 min after the sample was moved to the analytical column. After the gradient was completed, the column was washed with 100% mobile phase B (MP-B) first and then reconditioned with 99% MP-A for 30 min. The effluents from the LC column were ionized by electrospray ionization by applying 1,800 V to the metal union between the column and the electrospray tip. Electrosprayed ions were introduced into the mass spectrometer (Orbitrap Fusion Lumos) via a heated capillary (5.8 cm long with a rectangular slit of 1.6 mm long and 0.6 mm wide) maintained at 275°C for ion desolvation. The resulting ions were mass-analyzed by the Orbitrap at a resolution of 60,000 covering the mass range from 400 to 1,600 Da with a maximum injection time of 50 ms and automated gain control (AGC) setting of 2e5 ions. Mass spectra were recorded in the profile mode. Most abundant ions were subjected to MS2 analysis using the top speed mode, acquiring as many dependent scans as possible in the 3 s cycle time. The parameters used for these analyses were as follows. For MS2, ions with charge states from 2 to 8 were isolated by using a quadrupole mass filter in the monoisotopic peak selection mode using an isolation window of 1.2 Da, maximum injection time of 100 ms with AGC setting at 5e4 ions, and fragmented by high-energy collision dissociation (HCD) with nitrogen at 30% normalized collision energy. Fragment ions were mass analyzed by the Orbitrap at a resolution of 30,000, and spectra were recorded in the centroid mode. Ions once selected for MS2 were dynamically excluded for the next 60 s.

#### Proteomics data analysis

Raw MS data were searched against protein fasta files generated from the sequenced genomes of the MC-2 consortium (10) plus bovine trypsin and human keratin sequences to assess common contaminants using MaxQuant (34). Dynamic modification search included oxidized methionine and N-terminal acetylations. Static modification searches included alkylated cysteines. Parent ion tolerance was set to 20 ppm and considered only fully tryptic peptides. Label-free quantitation (LFQ) and iBAQ (but no match between runs) were selected as peptide identification parameters in MaxQuant. Peptides were filtered based on a 1% false discovery rate (FDR) using a decoy database.

Peptide abundance values for each sample were log2-transformed and mean-centered normalized. Peptides were rolled up to a protein value by summing the antilog values of each normalized peptide value. For the summed protein values used in the total protein and relative protein comparisons between the eight consortium member species, peptides used were those unique to a single protein derived from a single bacteria consortium member. For relative genus compositions calculated for each sample, total protein abundance values were summed across each genus and normalized to a percent value. For heatmaps displaying abundance and abundance fold-change values of individual proteins in the chitin degradation pathway, all peptides detected were used.

### Metabolomics analyses

#### LC-MS/MS metabolite analyses

LC-MS/MS runs were performed using a Hypersil Gold C18 reverse-phase column (150 × 2.1 mm, 3 µm particle size) (Thermo Scientific, Waltham, Massachusetts, USA) maintained at 40°C in a UHPLC Waters Acquity (Waters, Milford, Massachusetts, USA) coupled to a high-resolution Q-Exactive HF-X Orbitrap mass spectrometer (HRMS) (Thermo Fisher Scientific, Waltham, MA). Mobile phases consisted of the following: (A) 0.1% formic acid in water and (B) acetonitrile with 0.1% formic acid. The injection volume was set at 10 µL. At a flow rate of 0.4 mL/min, the elution initiated at 90% A (10% B) and was maintained for 2 min. The gradient thus linearly ramped up to 10% A (90% B) for the following 9 min, and those conditions were maintained for 1 min before the flow rate was increased to 0.5 mL/min for the following 30 s (min 12.5). The elution was maintained at those conditions for 1 min, and thus the gradient returned to the initial mobile phase proportions (90% A: 10% B) until 14 min of the chromatographic separation. The initial flow rate of 0.4 mL/min was linearly recovered for the following 30 s (min 14.5), and those conditions were maintained for an additional 30 s (min 15).

The HRMS operated in the data-dependent acquisition mode (DDA), and data were acquired in both positive and negative ionization modes. MS1 data were acquired using a Fourier Transform Mass Spectrometry (FTMS) full-scan mode for mass-to-charge ratio (*m*/*z)* ranging between 80 and 800 at a resolving power of 240,000. Maximum injection time (IT) was set at 200 ms and automatic gain control (AGC) at 3 × 10^6^ ions. MS2 data were acquired for the top 12 most intense ions for each scan at the centroid mode at a resolving power of 17,500. The MS2 parameters were set as follows: ion isolation window of 1.3 *m*/*z*, dynamic exclusion of 60 s, IT of 100 ms, AGC of 1 × 10^5^ ions, and collision energy of 20%, 30%, and 40%. A standard in-house mixture of metabolites was run as a quality control (QC) with every 20 samples.

#### Metabolite RAW data processing, Calibration, Filtering, and Completion

LCMS RAW files acquired in positive and negative ionization modes were processed using MZmine 3.2.3 (35, 36). Briefly, baselines of chromatograms were corrected, and ion masses and metabolomic features were detected (37), deconvoluted, aligned, and matched against an in-house LCMS metabolite library based on retention time (RT) and exact *m*/*z* values, corresponding to a second level of putative metabolite identifications (38). The peak areas of all detected MS1 peaks (metabolomic features) were exported to a CSV file, and *m*/*z* was subsequently calibrated through external and internal calibration steps. MS2 data were independently processed and exported to an MGF file and exported to SIRIUS 4 for further compound identification and classification.

#### Metabolite data set filtering

LCMS data sets were filtered at the cell level to reduce the amount of noise signals and non-representative data (i.e., features detected in only one or two samples), which commonly emerge from the mass spectrometry analyses. A cell includes all the replicate samples from the combination of all levels resulting from the different categorical factors of the experimental design: eight in our case [Control-0.weeks, Control-4.weeks, Control-8.weeks, Control-12.weeks; MSC2-0.weeks (0 wk), MSC2-4.weeks (4 wk), MSC2-8.weeks (8 wk), and MSC2-12.weeks (12 wk)]. The data filtering was performed via the following four main steps:

- Blank threshold. For a given variable, when the number of experimental blanks with data (>0) was equal to or lower than 30% of the total (2 out of 6 blank replicates), data for all experimental blanks for such a variable were considered 0.
- Signal to noise (*S/N*). Metabolite features with S/*N* < 10 across all cells were removed from the data set, and only those features in one or more cells with an average value at least 10 times the average of the experimental blanks were kept. The noise level was calculated for each feature individually from the six experimental blanks.
- Zero filter. To avoid spontaneous data in a given variable, which typically are shown in very low relative abundances, for each variable independently, when a cell had <30% of replicates with data, all values of those replicates were considered 0.
- Minimum data. Metabolite features with data in less than 50% of the replicates across all cells were removed from the data set. Only those variables with at least one cell containing data for at least 50% of the replicates were maintained in the data set.

#### Molecular formula assignment in MFAssignR

Molecular formulas were assigned to detected *m*/*z* signal values using the MFAssignR R package (39). Allowed molecular formulas were assigned according to the following constraints: (i) H/C and O/C ratios were set in agreement with the limits established previously (40), but with lower H/C ratios. Molecular formulas were thus constrained between 0.2–3 H/C and 0.01–1.2 O/C. (ii) Max number of heteroatoms, other than oxygen, was constrained as follows: *N* = 5, P = 1, and S = 2. (iii) Maximum ion charge was set at 1. (iv) Maximum formula matching error was set to 1 ppm. (v) Allowed DBE minus oxygen (DBE-O) was set between −13 and 15, a minor extension to that proposed in other studies (41). As a result, 99% of the assigned molecular formulas had DBE-O ranging between −7 and 10, with −4 and 7 being the 5th and 95th percentiles, respectively, in agreement with prior studies (41).

### Statistical analyses

Principal component analyses (PCAs) were used to visualize the overall similarities between samples for each of the data sets (species composition (16S), transcripts, proteins, and metabolites) through score plots of the two first principal components (Fig. 1b, 2b, 3b, 4d). The PCAs were performed in R (R Core Team 2021) (42) using imputePCA and PCA functions of the “missMDA” (43) and “FactoMinR” packages (44), respectively. One-way ANOVA on the score coordinates of the PCAs was performed to test for clustering significance between incubation periods using the aov function of the “Stats” package (R Core Team 2021). Pearson’s correlation coefficient was used to determine co-abundances between species based on 16 s analysis.

The Euclidean distances, as a proxy of the similarity between samples, were calculated for all data sets (species composition (16S), transcripts, proteins, and metabolites). Distances between consecutive incubation times (0 versus 4 weeks; 4 versus 8 weeks; 8 versus 12 weeks) were submitted to one-way ANOVA to test whether the magnitude of the overall changes across time points remained constant.

Gene expression and the relative abundance of enzymes from the chitin degradation pathway (KEGG map520), as well as their fold change between consecutive incubation times, were represented with heatmaps using the heatmap.2 function of the “gplots” package (Fig. 5).

LC-MS metabolite fingerprints of soil samples were submitted to hierarchical clustering analyses for both metabolite features and samples to obtain a global visualization of the metabolomic fingerprints with the heatmap.2 function of the “gplots” package (Fig. 4a). Soil samples that grouped in the dendrograms had more overall metabolite similarities between them. Similarly, variables that were closely grouped in the metabolic feature dendrograms indicated similar patterns, in terms of relative abundances, across all soil samples. A heatmap was constructed to represent the relative abundances of individual metabolomic features that were grouped into different clusters on the dendrograms. Data for each metabolic variable were scaled from 0 to 1, with 1 representing the maximum relative abundance across all samples. Hierarchical clustering analyses were computed using the Euclidean distances following the complete method. In addition, metabolomic fingerprints of consecutive soil incubation periods were submitted to permutational multivariate analysis of variance (PERMANOVA) for testing overall differences between them (Tables S9 and S10). The relative intensities of metabolite features assigned to molecular formulas and subsequently classified into the different overall compound classes (amino sugars, oxyaromatic compounds, protein, lipids, and carbohydrates) were added up into a single variable for each compound class. This new data set was submitted to PCA using the ggbiplot function of the “ggbiplot” package (45) and a biplot (scores and loadings) to visualize the overall weight of the different compound classes across soil samples (Fig. 4e). The relative abundances of all features assigned to each of the overall compound classes were summed to obtain a single value of each compound class per sample. Those values, representing the total abundances of amino sugars, oxyaromatic compounds, protein, lipids, and carbohydrates, were submitted to one-way ANOVA to test for differences between incubation times.

Individual peptide and metabolomic features were submitted to one-way ANOVA with incubation time (0 weeks, 4 weeks, 8 weeks, and 12 weeks) as categorical factors. Tukey HSD tests were subsequently used for each individual test at a significance level of 0.05. Tukey HSD tests were performed using the HSD.test function of the “agricolae” package (46).

## Data Availability

Mass spectrometry raw instrument data and analyzed data sets for proteome, GC-MS metabolome, and LC-MS metabolome are publicly available through MassIVE accession MSV000090377 at https://massive.ucsd.edu. RNA-Seq data are publicly available from the Gene Expression Omnibus through accession number GSE251759. 16S rRNA amplicon data and Data sets S1 to S3 are publicly accessibly on PNNL’s DataHub at https://data.pnnl.gov/group/nodes/dataset/34237.

## SUPPLEMENTAL MATERIAL

Fig. S1 mbio.00404-25-s0001.pdf Experimental protocol.

Fig. S2 mbio.00404-25-s0002.pdf DNA amounts.

Fig. S3 mbio.00404-25-s0003.pdf Relative abundances of inoculum at time 0.

Fig. S4 mbio.00404-25-s0004.pdf Relative abundances over time.

Fig. S5 mbio.00404-25-s0005.pdf Absolute values of 16S-based abundances.

Fig. S6 mbio.00404-25-s0006.pdf Absolute metatranscriptomics counts.

Fig. S7 mbio.00404-25-s0007.pdf Total protein abundances.

Fig. S8 mbio.00404-25-s0008.tif Shifts in metabolite groups across time.

Fig. S9 mbio.00404-25-s0009.pdf PCA analyses.

Supplemental Tables mbio.00404-25-s0010.xlsx Tables S1-S10.

ASM does not own the copyrights to Supplemental Material that may be linked to, or accessed through, an article. The authors have granted ASM a non-exclusive, world-wide license to publish the Supplemental Material files. Please contact the corresponding author directly for reuse.
