# Supplementary material for: Multi-omics of a model bacterial consortium deciphers details of chitin decomposition in soil
Source: mBio. 2025 May 30;16(7):e00404-25. doi: 10.1128/mbio.00404-25 (PMC12239585; doi:10.1128/mbio.00404-25)
Supplement: Fig. S1 — Experimental protocol. [file mbio.00404-25-s0001.pdf]

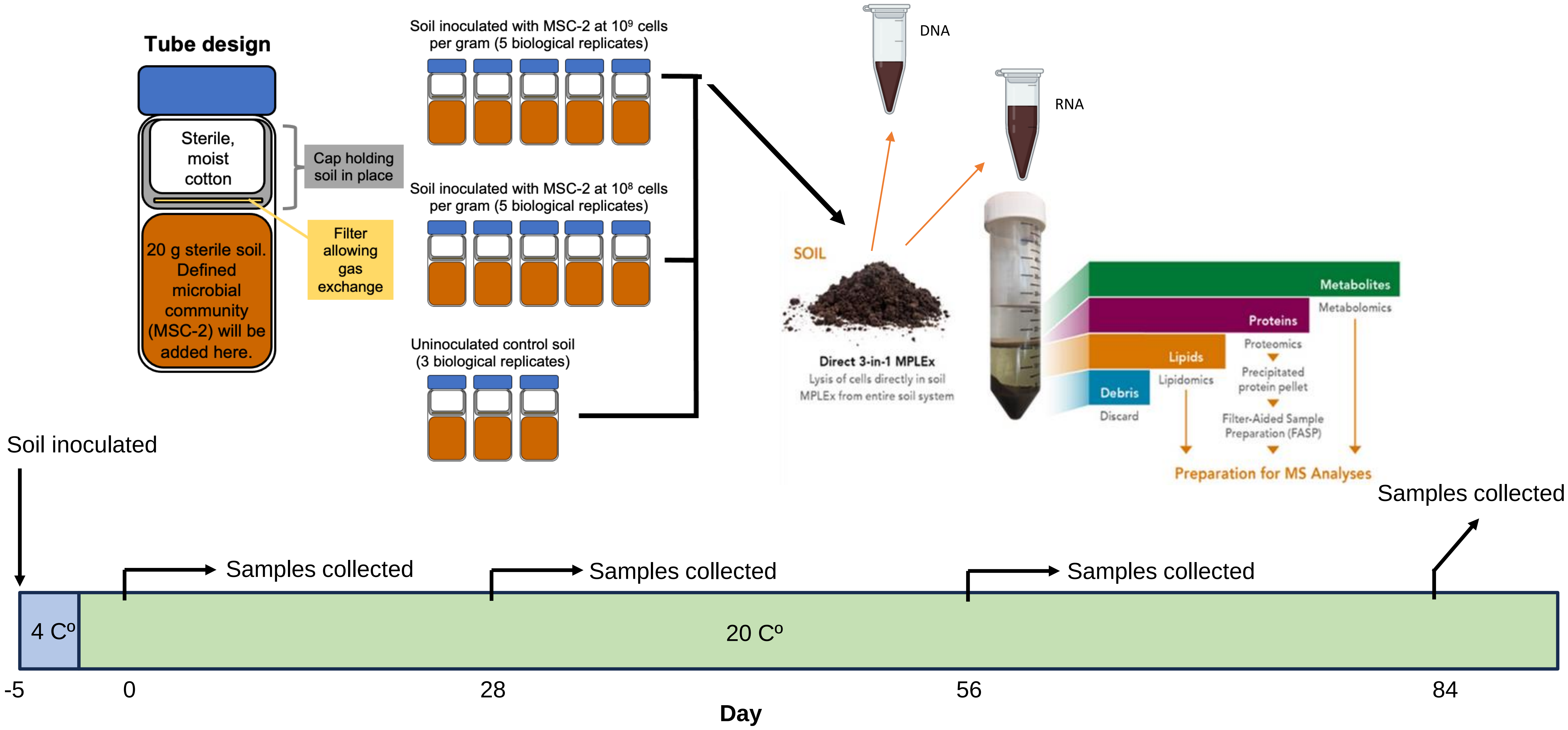


**Supplementary Figure 1.** Tube design as well as an experimental schematic and isolation protocol are shown. Tubes of soil were inoculated at day -5 and allowed to incubate at 4 C° for 5 days before being moved to 20 C°. At 0, 28, 56 and 84 days post shifting to 20 C samples were collected for multi-omic analysis.
