# Supplementary material for: Multi-omics of a model bacterial consortium deciphers details of chitin decomposition in soil
Source: mBio. 2025 May 30;16(7):e00404-25. doi: 10.1128/mbio.00404-25 (PMC12239585; doi:10.1128/mbio.00404-25)
Supplement: Fig. S2 — DNA amounts. [file mbio.00404-25-s0002.pdf]

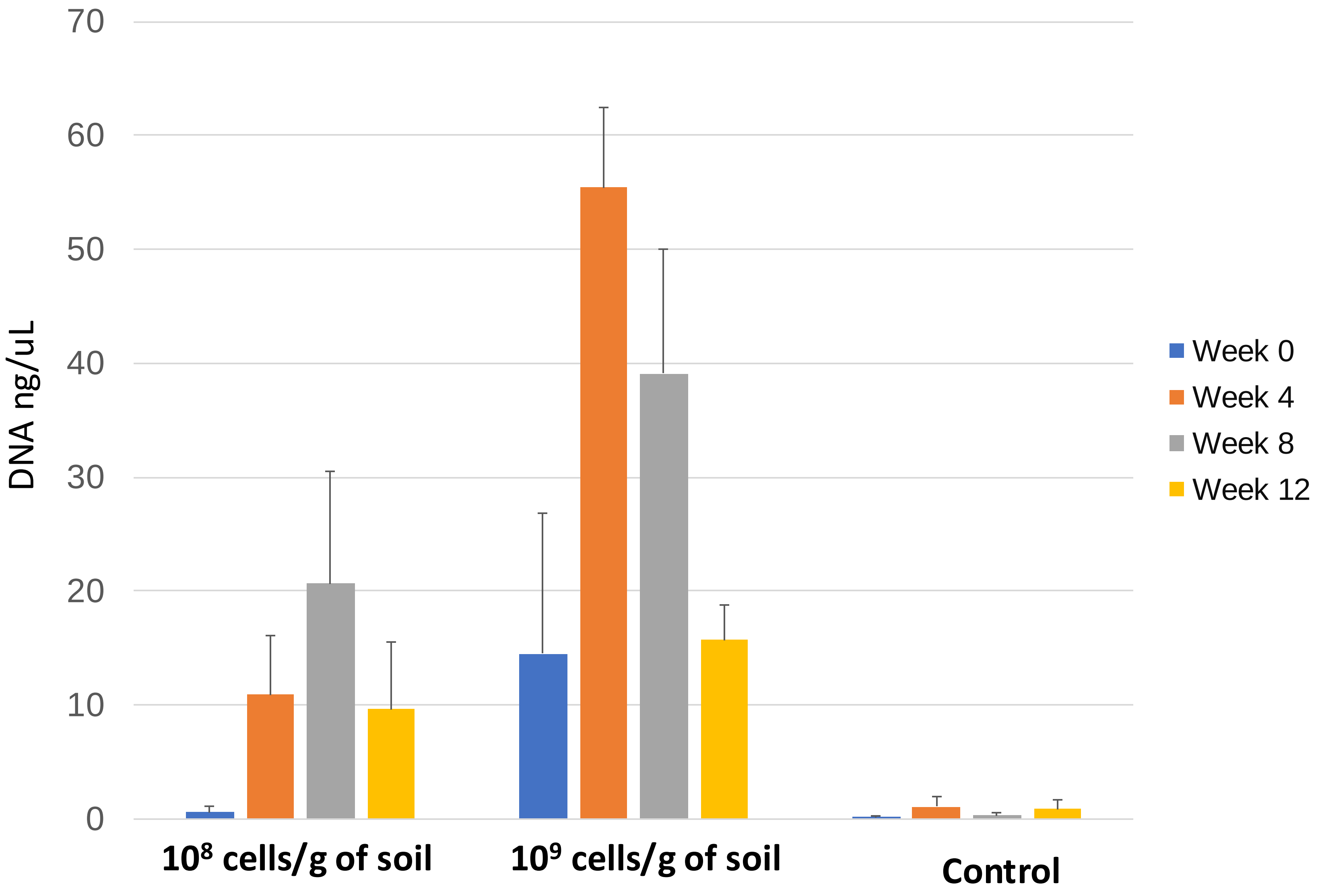

Supplementary Figure 2. DNA amounts in MSC-2 inoculated soil. Amount of DNA is shown on the y-axis and weeks are shown as separate-colored bars: Time 0, blue; Week 4, orange; Week 8, grey; Week 12 yellow. Bars are grouped by inoculation amount (10^8 cells gram^{-1} of soil, 10^9 cells gram^{-1} of soil and Control (no inoculation).
