# Supplementary material for: Multi-omics of a model bacterial consortium deciphers details of chitin decomposition in soil
Source: mBio. 2025 May 30;16(7):e00404-25. doi: 10.1128/mbio.00404-25 (PMC12239585; doi:10.1128/mbio.00404-25)
Supplement: Fig. S3 — Relative abundances of inoculum at time 0. [file mbio.00404-25-s0003.pdf]

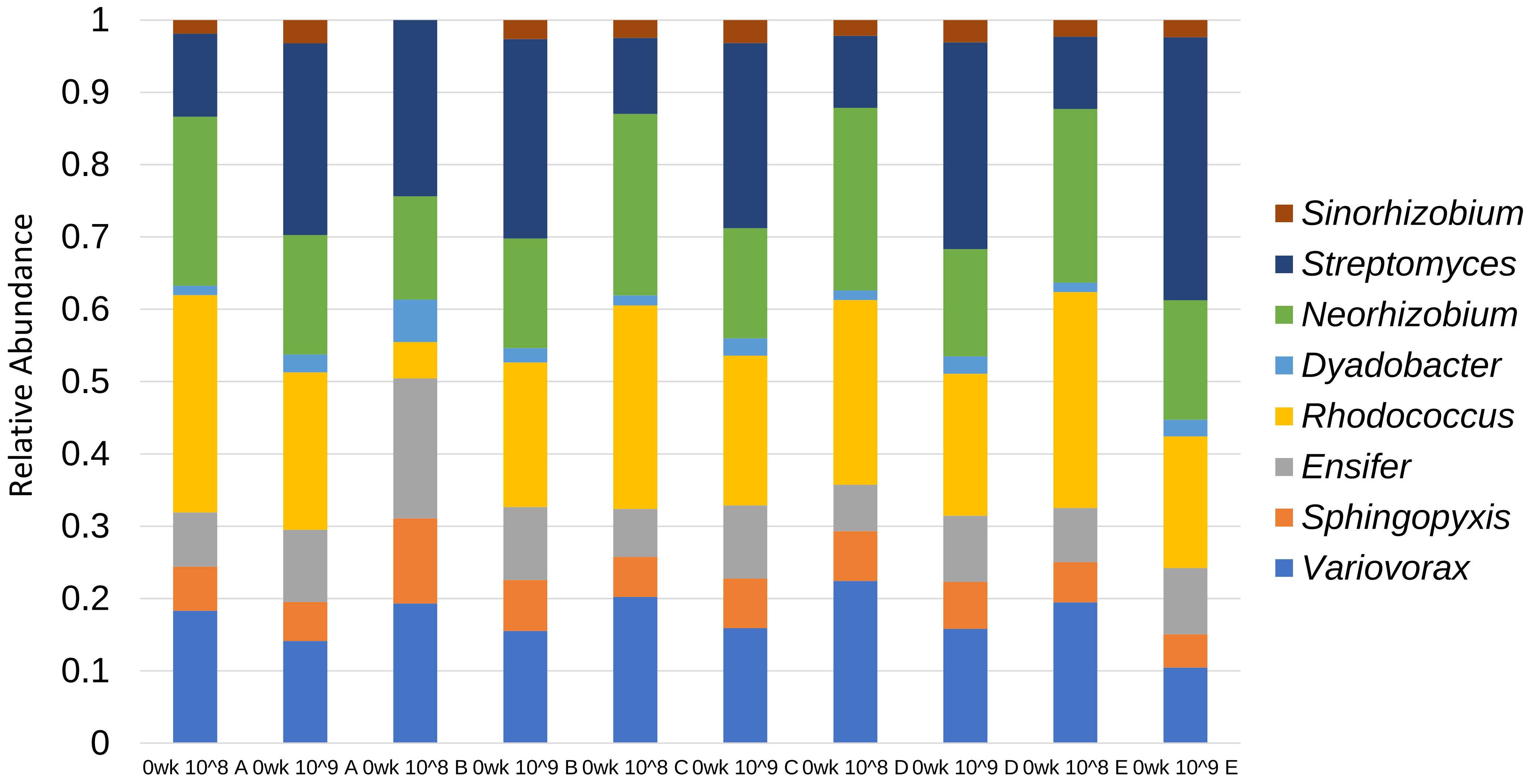

Supplementary Figure 3. Relative abundances of MSC-2 members at initial timepoint. Timepoints, replicates and concentrations of cells are shown on the x-axis (e.g. 10^9 indicates soil inoculated with 10^9 cells per gram of soil). Relative abundances are shown in the y-axis with members colors shown on the right.
