# Supplementary material for: Multi-omics of a model bacterial consortium deciphers details of chitin decomposition in soil
Source: mBio. 2025 May 30;16(7):e00404-25. doi: 10.1128/mbio.00404-25 (PMC12239585; doi:10.1128/mbio.00404-25)
Supplement: Fig. S4 — Relative abundances over time. [file mbio.00404-25-s0004.pdf]

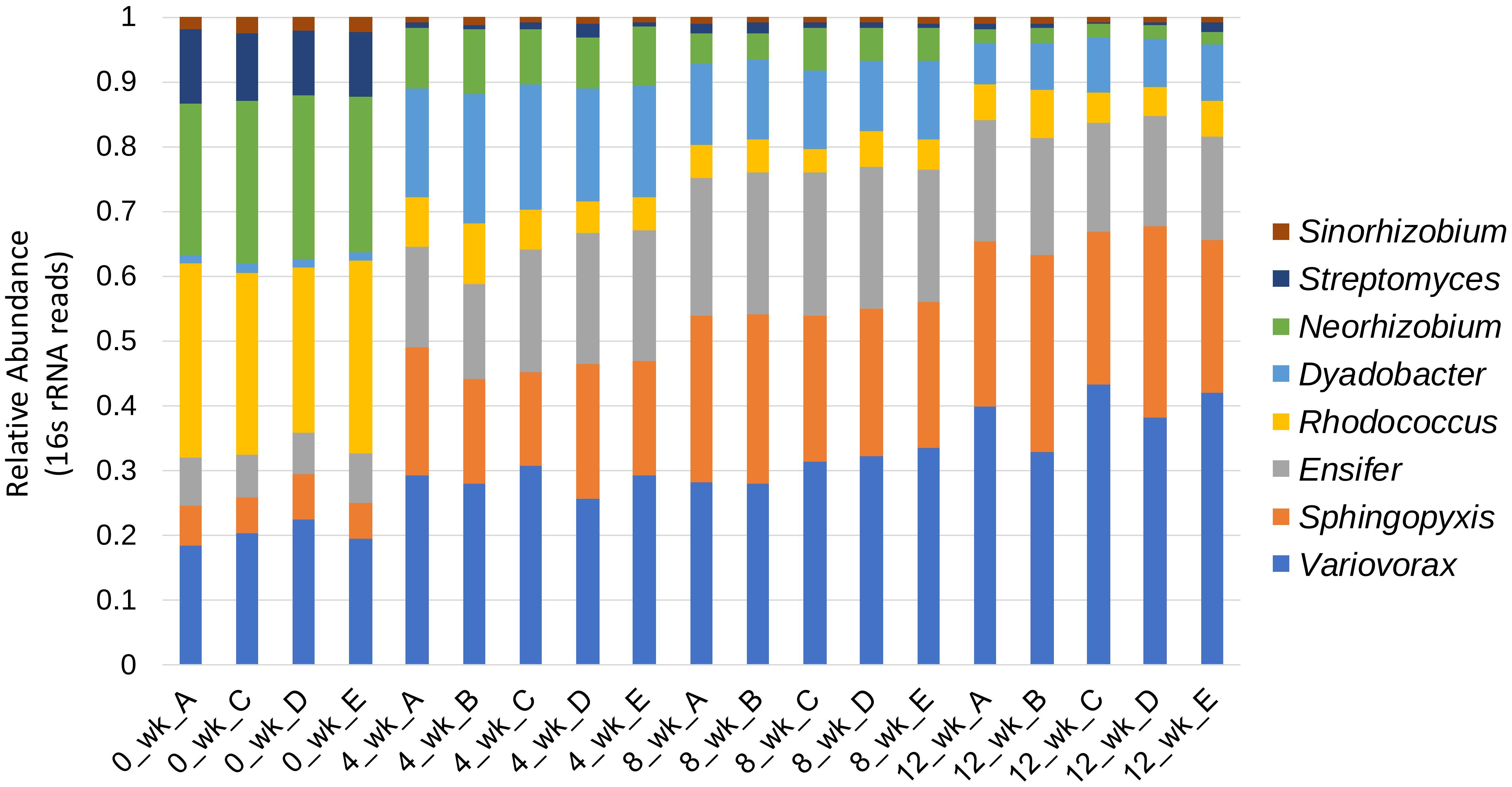

Supplementary Figure 4. Relative abundances across time for samples inoculated with the lower inoculum dose (108 cells gram^{-1}). Relative abundance is shown in the y-axis. Timepoints and replicates are shown in the x-axis. All samples are from soil inoculated with 10^8 cells per gram of soil. Member species are indicated by color (right panel).
