# Supplementary material for: Multi-omics of a model bacterial consortium deciphers details of chitin decomposition in soil
Source: mBio. 2025 May 30;16(7):e00404-25. doi: 10.1128/mbio.00404-25 (PMC12239585; doi:10.1128/mbio.00404-25)

Supplementary Figure 5. Absolute value 16S rRNA based abundance of MSC-2 species over 0, 4, 8 and 12 weeks of incubation. Letters below each column correspond to one of five sample replicates.

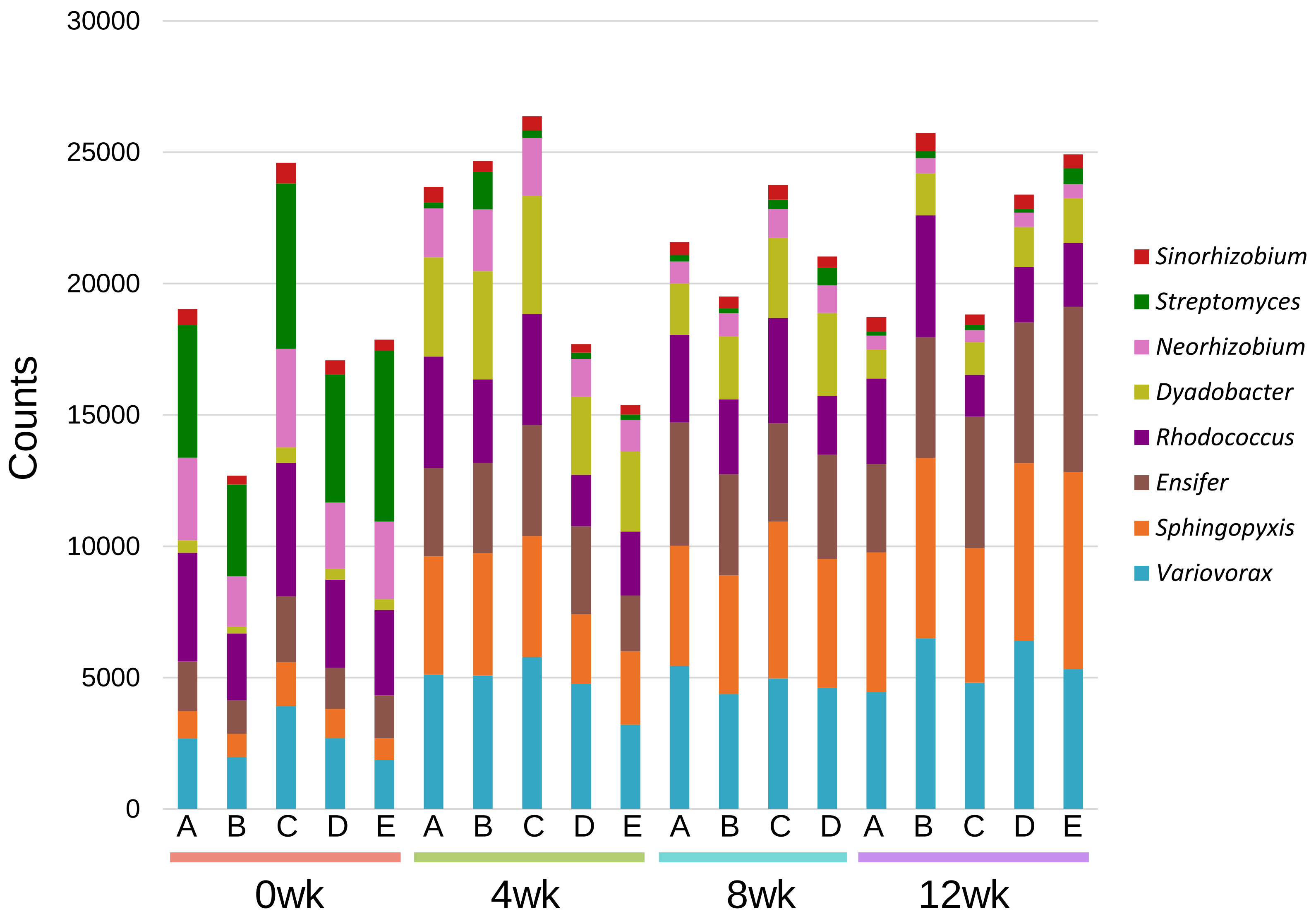

Supplement: Fig. S5 — Absolute values of 16S-based abundances. [file mbio.00404-25-s0005.pdf]
