# Supplementary material for: Multi-omics of a model bacterial consortium deciphers details of chitin decomposition in soil
Source: mBio. 2025 May 30;16(7):e00404-25. doi: 10.1128/mbio.00404-25 (PMC12239585; doi:10.1128/mbio.00404-25)

Supplementary Figure 6. Absolute metatranscriptomic counts for each species. Number of counts was normalized to the size of the genome. Letters below each column correspond to one of five sample replicates at 0, 4, 8 and 12 weeks.

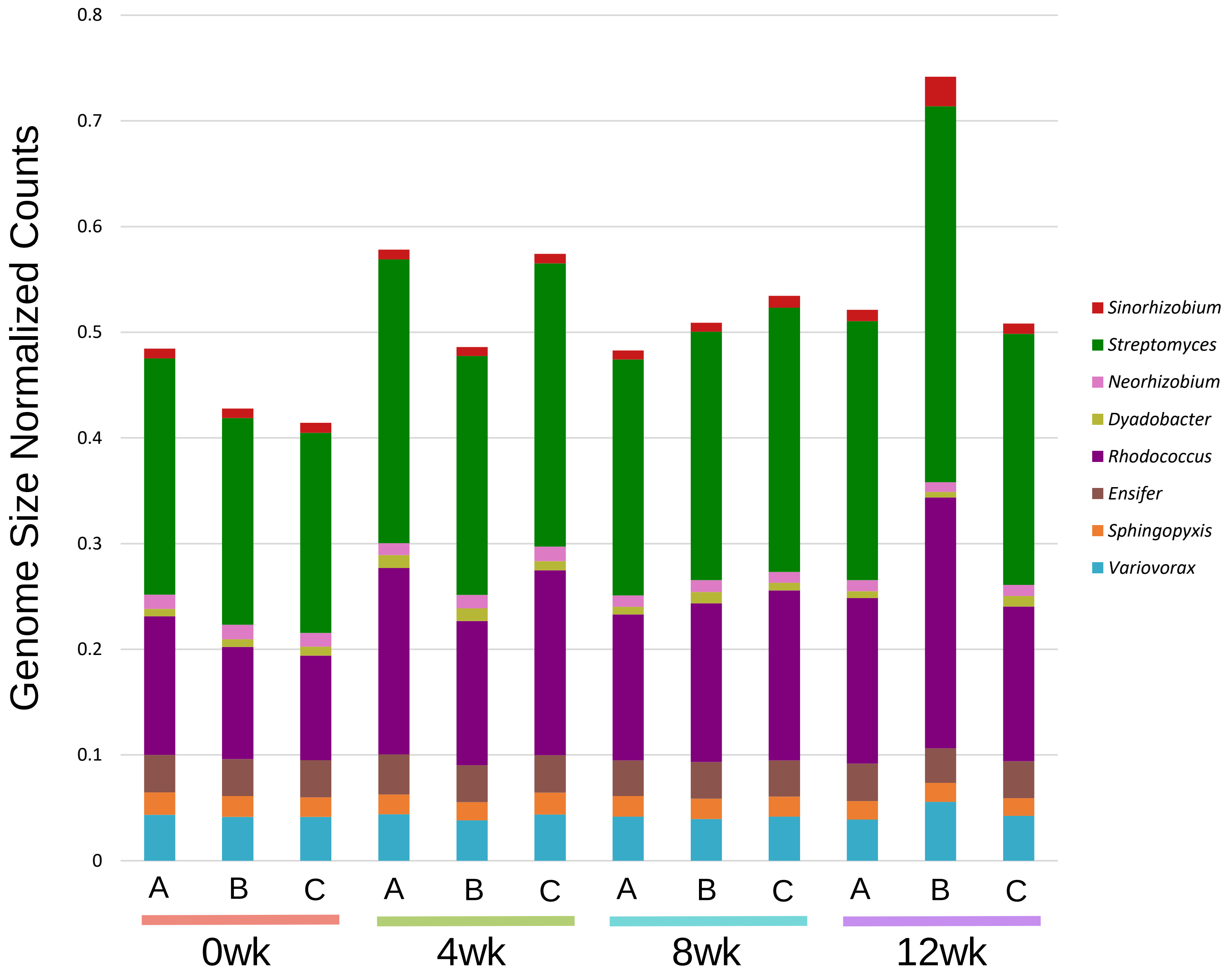

Supplement: Fig. S6 — Absolute metatranscriptomics counts. [file mbio.00404-25-s0006.pdf]
