# Supplementary figures and images for: Multi-omics of a model bacterial consortium deciphers details of chitin decomposition in soil
Source: mBio. 2025 May 30;16(7):e00404-25. doi: 10.1128/mbio.00404-25 (PMC12239585; doi:10.1128/mbio.00404-25)

**Supplementary Figure 7.** Total Protein Abundances for MSC-2 Species in Soil

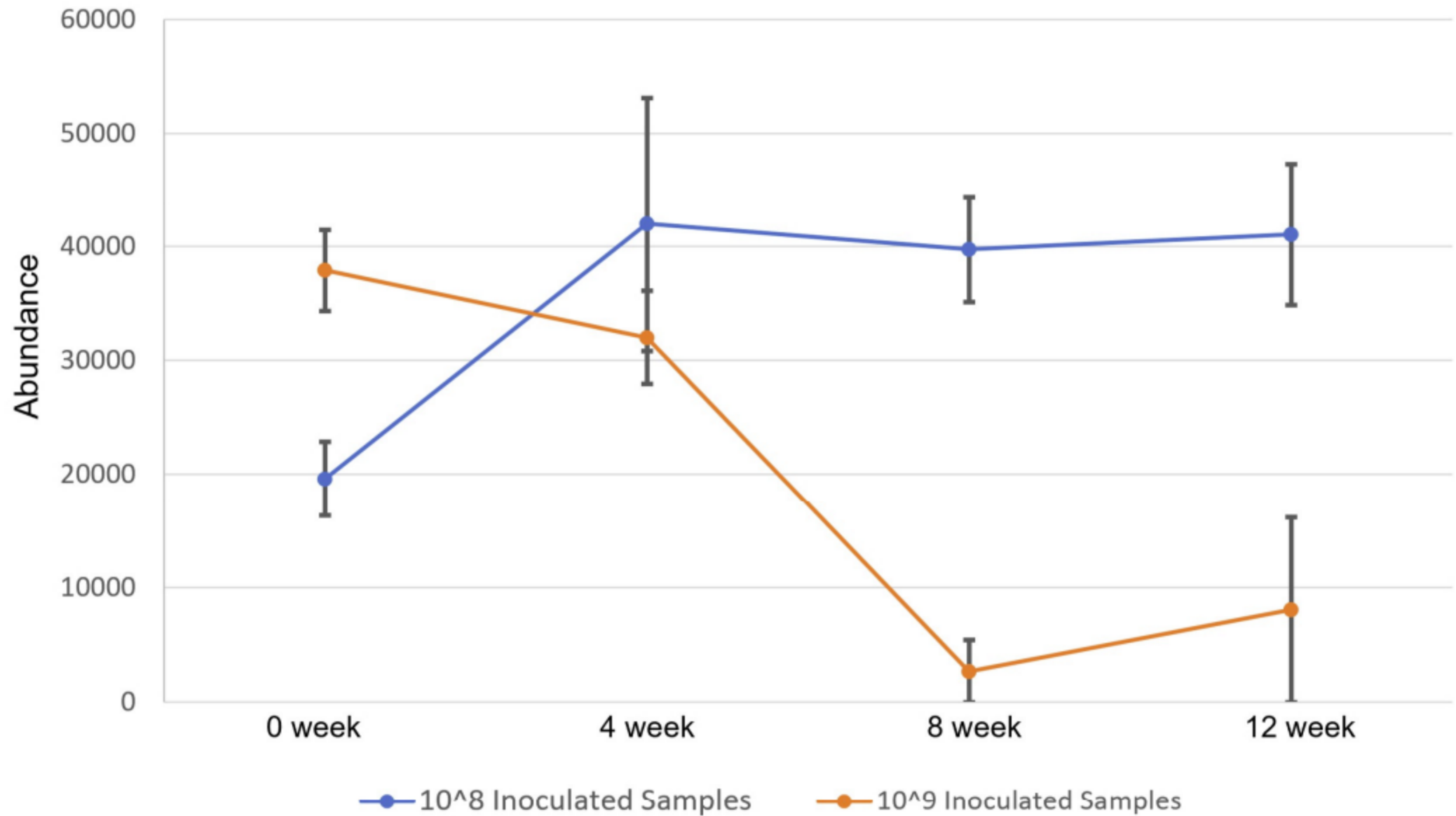

Supplement: Fig. S7 — Total protein abundances. [file mbio.00404-25-s0007.pdf]

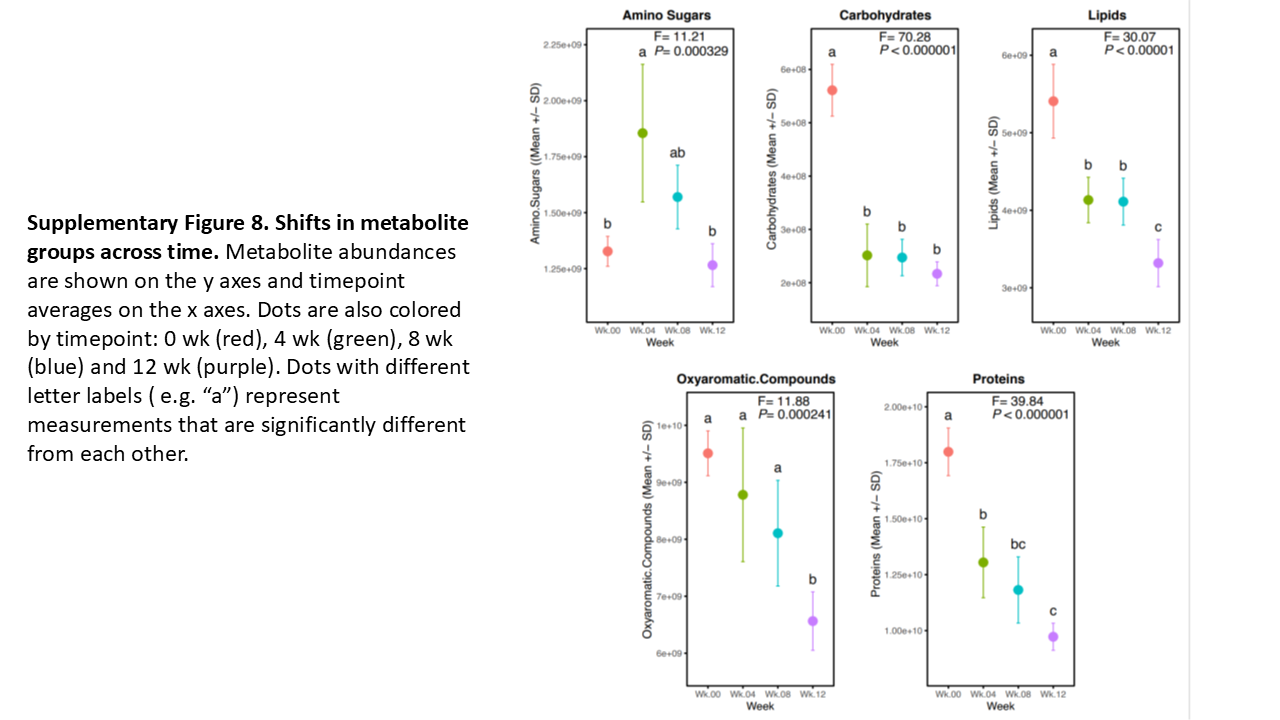

Supplement: Fig. S8 — Shifts in metabolite groups across time. [file mbio.00404-25-s0008.tif]
