# Supplementary material for: Multi-omics of a model bacterial consortium deciphers details of chitin decomposition in soil
Source: mBio. 2025 May 30;16(7):e00404-25. doi: 10.1128/mbio.00404-25 (PMC12239585; doi:10.1128/mbio.00404-25)
Supplement: Fig. S9 — PCA analyses. [file mbio.00404-25-s0009.pdf]

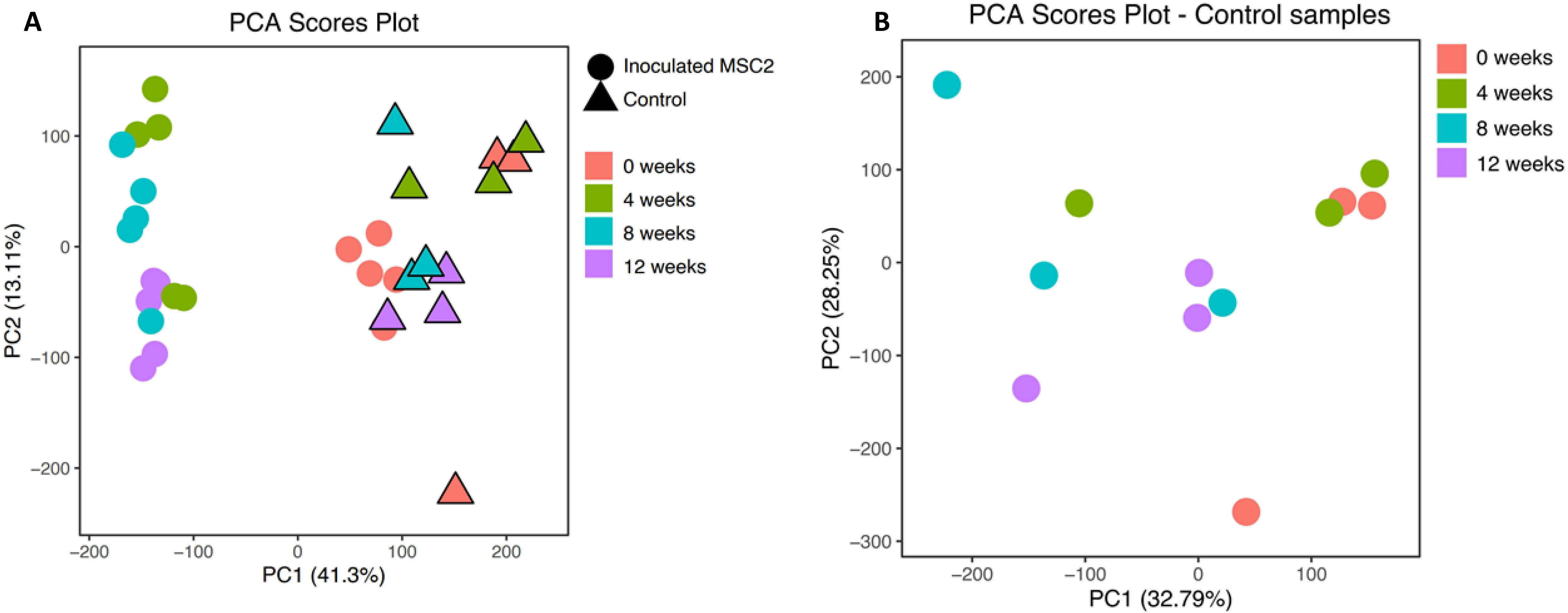

Supplementary Figure 9. PCA analyses of metabolite data. a) Principal component (PC) 1 vs. PC2 of the score plot of the principal component analysis (PCA) of metabolite data from control (triangles) and inoculated samples (circles) at 0, 4, 8, and 12 incubation weeks. **b)** Score plot of the PCA including solely the metabolite data of control samples across all incubation weeks. Different colors indicate different incubation weeks (red: 0 weeks, green: 4 weeks, blue: 8 weeks, purple: 12 weeks).
